# Supplementary material for: Epigenome-wide association meta-analysis of DNA methylation with coffee and tea consumption
Source: Nat Commun. 2021 May 14;12:2830. doi: 10.1038/s41467-021-22752-6 (PMC8121846; doi:10.1038/s41467-021-22752-6)
Supplement: Supplementary file 1 — Supplementary Information [file 41467_2021_22752_MOESM1_ESM.pdf]

Supplementary Methods

Description of participating cohorts

ALSPAC Study: ALSPAC stands for Avon Longitudinal Study of Parents and Children, an ongoing epidemiological study of children born from 14,541 pregnant women residing in Avon, UK. Methylation data from mothers was estimated from DNA extracted from blood, while from children from cord blood. For the purpose of our study, only mothers participated. More extensive details of the available data can be assessed through a study website with a fully searchable data dictionary (http://www.bris.ac.uk/alspac/researchers/data-access/data-dictionary/), while details regarding study itself can be retrieved elsewhere [1, 2]. ALSPAC participants were asked how many cups of coffee and tea they currently drank (open format) separately for weekdays and weekends. The replies to these questions were recoded into one measure of daily use in cups/day. In ALSPAC cohort, the smoking was either never or ever smokers (current and former combined). The BMI was computed as it is common. The data on coffee and tea consumption was collected several years prior to the DNA methylation data. Since the coffee/tea consumption tends to be stable over longer period of time [3, 4], beverage consumption data was used from time point several years prior to the DNA methylation data. Time difference adjustment was additionally added in the model due to the potential confounding affect that the time might have had on the exposure variable. All the other covariates were collected at the same time point as DNA methylation data.

Airwave Study: The Airwave Health Monitoring Study is an occupational cohort consisting of 28 police forces employees from Great Britain. More details of the Airwave cohort can be found elsewhere [5]. Cohort was set up to assess the possible health risks associated with the use of TETRA (digital communication system), used by the police forces and other emergency services. The study officially started in 2004 with 53,280 participants first recruited with current response rate estimated at 53,265. At the baseline recruitment, participants completed enrolment questionnaire, or health screening or both. Blood samples were spun at the health clinic while the biological samples were stored in a Thermoporter (LaminarMedica) and for long terms storage frozen at -80°C. Blood was used for DNA extraction and subsequently DNA methylation levels estimates. For the analysis, coffee and tea consumption was collected via self-reported survey Alcohol was defined as never drinker, current drinker and ex-drinker and treated as categorical variable in the analysis. At the participants enrolment, height and weight data was collected via standardized protocol, from which body mass index (BMI) was computed as a ratio between weight (kg) and height (m^2).

ARIC Study: The Atherosclerosis Risk in Communities (ARIC) study is a prospective cohort, originally established to assess the etiology of atherosclerosis and subsequently medical care, cardiovascular risk factors et cetera. Participants were recruited from four US communities where in total, 7082 men and 8710 women were recruited (age 45-64). More details of the cohort description can be found elsewhere [6]. Participants underwent initial baseline examination and were followed up in six subsequent exams. In order to investigate potential contribution of ethnicity on dietary and/or methylation changes, the participants who had DNA methylation and FFQ data available were split in 2 cohorts based on their ethnicity (European n=1,099; and African American n=2,736). The data on coffee and tea consumption were collected as follows: average coffee intake over the past year with choice responses: almost never, 1-3 cups/month, 1 cup/week, 2-4 cups/week, 5-6 cups/week, 1 cup/day, 2-3 cups/day, 4-5 cups/day and 6+ cups/day. All the other covariates were collected either via questionnaire

or physical examination. In the model, additional surrogate variables were used to correct for batch effects.

CHS Study: The cardiovascular Health Study (CHS) is an ongoing population-based cohort study initiated to study risk factors involved in stroke and coronary heart disease in adults ≥65 years of age recruited at four centres across the US. Initially, participants recruited were of primarily European ancestry (n=5,201), and participants of African American ancestry were later recruited in the study (n=687). For our analysis, participants having both DNA methylation and coffee/tea consumption data provided 195 participants of European and 185 participants of African American ancestries, where the association was performed separately for each ethnicity. Dietary data were collected using a picture-sort version of the National Cancer Institute food frequency questionnaire (FFQ) [7]. Based on the previous year, participants were asked to select one of the following answers for each item in the FFQ: never or < 5 times a year, 5-10 times a year, 1-4 times a month, 1- 4 times a week and almost every day. The data on coffee and tea consumption were collected several years prior to the blood draw for the methylated DNA, hence additional time-difference adjustment was introduced in the model, while all the other covariates were collected at the same time as blood draw for the methylated DNA.

EPIC Study (Epic_Italy and Epic_IARC): The European Prospective Investigation into cancer and Nutrition (EPIC) is a study with over 521 000 participants enrolled from 23 centres and among 10 western European countries [8]. The study participants have had biological samples collected including plasma, serum leukocytes and erythrocytes at baseline and is one of the biggest biobanks in the world. At the recruitment, detailed information such as anthropometric measurements, lifestyle characteristics, diet and medical history were collected. The blood for DNA methylation was extracted using QIAmp Blood Mini Kit (QIAGEN). Among other covariates, Epic_IARC (n=866) cohort additionally adjusted for surrogate variable to control for batch effects. For Epic_IARC cohort data on coffee consumption was collected via food frequency questionnaire, while in Epic_Italy (n=1,096), data on coffee consumption was collected via questionnaire. The Epic_IARC cohort includes prospective breast cancer cases and matched controls and EPIC_Italy had in total 3 case-control studies nested in the study on breast cancer, lung cancer and colorectal cancer, for which they adjusted additionally in cohort specific adjustment. Furthermore, in Epic_Italy cohort participants were recruited in different cities (Florence, Milan, Naples, Ragusa, and Turin) and this information was used as an additional adjustment variable (treated as random effect).

ESTHER Study (Esther_a, Esther_b): Esther study is an ongoing population-based cohort where participants are recruited from the federal state of Saarland, Germany. In summary, 9,949 participants were recruited via their general practitioner (GP) and underwent routine health check-ups, as well as collection of epidemiological data (socio-demographic characteristics, history of major diseases and lifestyle factors). Data on previously mentioned variables was collected by self-administered questionnaire completed by participants, while the biological samples (urine, stool and blood) were obtained and stored at -80°C. DNA was extracted from blood and DNA methylation levels were determined by Illumina HumanMethylation 450K array. Coffee and tea consumption were obtained from the FFQs as following: 1=several times per day, 2=once per a day, 3=several times per week, 4=once a week, 5=less than once a week and 6=never, from which the average intake was calculated (cups/day). Other confounding variables (e.g. alcohol, BMI, smoking) were collected either via questionnaire or during health check-ups, where ever smoking was defined as ≥100 cigarettes during his/her lifetime, thereby avoiding misclassification of rare occasional smoking. Ever smoker was defined as participant who quit for ≥1 year prior to the study collection. Two random subsets of ESTHER are referred to: Esther_a and Esther_b and analysis were performed separately, more details can be found elsewhere [9-11].

FHS Study: The Framingham Heart Study (FHS) is a community-based, ongoing cohort study which originally started in 1948. The original participants (n=5,209) were randomly recruited in the city of Framingham, MA, USA and follow up for every 2 years onward, since 1948. The children or participants and children's spouses were named as Offspring cohort (n=5,124) and were subsequently enrolled. DNA methylation from blood was assessed on 2,846 Offspring participants from 8th examination cycle (2005-2008), where DNA was extracted using Puregene DNA extraction kit (Qiagen, Venlo, Netherlands). Other covariates were collected either via questionnaire, measurement of anthropometric data and other more extensive collection procedures, more details regarding study can be found elsewhere [12]. Smoking was defined as ever and never (former and never combined) smokers. For coffee consumption data, participants had a choice to fill in either decaffeinated, coffee with caffeine or dairy coffee drink consumption (from which variable of coffee consumption was computed as: coffee with caffeine + ½ dairy coffee drink), while frequency of either beverages could be any of the following choices: 1-3 cups/month, 1 cup/week, 2-4 cups/week, 5-6 cups/week, 1 cup/day, 2-3 cups/day, 4-5 cups/day, 6+ cups/day. The average intake of cups/day was estimated where e.g. 2-3 cups would be treated as 2.5 cups/day.

KORA Study: The KORA (Kooperative Gesundheitsforschung in der Region Augsburg) research platform has been collecting clinical and genetic data from the general population in the region of Augsburg, Germany for over 20 years. F4 (2006-2008) and FF4 (2013-2014) cohorts are follow-up studies from the KORA S4 (n=4,261) survey carried out 1999-2000, KORA F3 is a ten years follow-up study of the KORA S3 (n=4,856) survey (1994-1995). In the baseline examinations all inhabitants of German nationality between the ages of 25 and 74 years were enrolled. Participants completed a lifestyle questionnaire, including details on health status and medication use, underwent standardized examinations with blood samples taken (PMID: 16032513). Genomic DNA extracted from blood was bisulfide-converted using EZ-96 DNA Methylation kit (Zymo Research) according to the manufactures protocol, while genome-wide DNA methylation was assed via Illumna HumanMethylation 450 BeadChip, following Illumina Infinium HD Methylation protocol. Coffee and tea consumption were collected as cups/day via questionnaire. The KORA cohort ethical approval was granted by the ethics committee of the Bavarian Medical Association and all were carried out in accordance with the principles of the Declaration of Helsinki. All research participants have signed informed consent prior to taking part in any research activities

LifeLines: Lifeline is a population cohort originating in the northern provinces of The Netherlands with an original aim to investigate association between healthy ageing and environmental, phenotypic as well as genomic factors. The study initiated from 2006 to 2019, where participants living at the northern part of The Netherlands were invited to participate together with their families and contribute to the 3 generation study design. All the participants underwent extensive measurements of metabolic and cardiovascular health, including anthropometry, detailed questionnaires, collection of blood samples and complete blood count. The average daily consumption of coffee (cups/day) was calculated as an average from questionnaire with following options: not this month, 1 day/week, 2-3 days/month, 2-3 days/week, 4-5 days/week, with an additional option of how often the coffee was consumed over the past month and on days the coffee was consumed, how many cups were consumed on average. Whole blood was stored at -80°C, from which the DNA methylation levels were measured.

RS Study: Rotterdam Study is a population based cohort where participants were recruited from the Ommord District, Rotterdam, The Netherlands. General design and overview of the study can be found described in more details elsewhere [13]. Briefly, participants were > 45 years underwent self-assessed questionnaires and physical exam. Whole blood was used for extraction of DNA, stored in EDTA tubes. Illumina HumanMethylation 450K array was used for determining the genome-wide DNA methylation

levels. Coffee and tea consumption data were collected from previously validated 389 item food-frequency questionnaire [14]. The data on tea consumption was collected on black, green and herbal. We combined black and green tea as previously explained in the Methodology section of the manuscript. For the purpose of this study, data was collected from two separate cohorts: The third visit of RS-II and the first and second visit of RS-III. In RS-III, a selection of participants had DNA methylation data collected at the first visit (RS-III-1) and other part (not overlapping) had DNA methylation data collected at second visit (RS-III-2). However, in RS-III-2 the data for coffee and tea consumption was collected several years prior to DNA methylation data (at RS-III-1 visit). As it is case for ASLPAC and CHS cohorts, difference adjustment was additionally added in the model and all the other covariates were collected at the same time point as DNA methylation.

TwinsUK Study: The TwinsUK registry was initiated in 1992 to recruit healthy participants who were either monozygotic or dizygotic same-sex twins, aged over 18 [15]. In total, there are more than 14,000 participants recruited across the UK, where the majority being adult female of European descent. The number of females who had both whole blood DNA methylation profiles and data on coffee and tea consumption for the purpose of this study was 552 subjects. DNA for methylation assessment was extracted from whole blood and stored in EDTA tubes. The Infinium HumanMethylation450 BeadCHips (Illumina Inc, San Diego, CA) was used to measure DNA methylation levels, as previously described [16]. Data on coffee and tea consumption in the TwinsUK cohort was collected following the EPIC-Norfolk guidelines [17]. Data on coffee and tea consumptions was collected using the following options: never or less than once a month, 1-3 per a month, once a week, 2-44 per a week, 5-6 per a week, once a day, 2-3 per a day, 4-5 per a day and 6+ per a day. These data were subsequently used to calculate the average intake per a day (cups/day).

Ethical approval was granted by the National Research Ethics Service London-Westminster, the St Thomas' Hospital Research Ethics Committee (EC04/015 and 07/H0802/84). All twins provided written informed consent prior to taking part in research activities.

DNA methylation profiling

All participating cohorts measured DNA methylation in peripheral blood using the Infinium Human Methylation 450K Bead-Chip (Ilumina, San Diego, CA, USA) except Airwave cohort, where the Infinium Methylation EPIC (850K) Bead-Chip was used [18]. Genomic DNA was bisulfite-converted for methylation measurement. Following bisulfite-conversion of DNA, samples underwent whole-genome amplification and were fragmented and hybridized on Bead-Chip with their complementary probe sequences. DNA methylation status was assessed through a single-base extension step. Arrays were imaged with a high precision scanner (iScan system, Illumina Inc.) and the signal intensities were extracted by usage of a software package (GenomeStudio Software, Illumina Inc.). DNA methylation status was calculated with the β value - signal from the methylated probe divided by the overall signal intensity. The methylation percentage of CpG sites was reported as a continuous β value range between 0 (no methylation) and 1 (full methylation). As commonly performed, DNA methylation data pre-processing was conducted independently in different cohorts and β values were normalized by study-specific methods. DNA methylation sites were annotated with the information provided by Illumina and the University of California Santa Cruz (UCSC) database (GRCh37/hg19).

EWAS of coffee and tea consumption

DNA methylation was considered as the dependent variable with coffee or tea consumption as predictors of interest (cups/day), each. Conventionally, each participating cohort performed an EWAS as a set of mixed effects liner-regression models, one CpG site at a time. In total, two linear mixed effects regression models were computed for each of the two exposures of interest. In the basic model (Model 1): we included age, sex, smoking status (never, former, and current), white blood cells (either measured directly or imputed based on Houseman algorithm [19]) as fixed effects and technical covariates as random effects to control for batch effects. In the second model (Model 2), we additionally adjusted for body mass index (BMI, kg/m^2) and alcohol consumption (g/day). All of the potential confounders were collected at the same time point of blood sampling for DNA methylation. Genetic principal components were included as covariates to account for population stratification if required.

For a subset of cohorts (ARIC_AA, ARIC_EA, ALSPAC, FHS, EPIC_IARC) surrogate variables were calculated and adjusted for in the modelling, due to the batch effects not controlled adequately by other modelling techniques. Airwave cohort did not have alcohol in gr/day, but the categorical variable (never drinker, current drinker, and ex-drinker) for which they adjusted in the analysis. FHS cohort had current and ever smokers (never and former combined), while ALSPAC cohort had only smoking variable defined as smokers (current and former combined) and non-smokers. The EPIC_IARC cohort includes prospective breast cancer cases and matched controls and EPIC_ITALY had in total 3 case-control studies nested in the study on breast cancer, lung cancer and colorectal cancer, and they adjusted for cases status. In addition, EPIC_ITALY had participants recruited from different cities in Italy, where center of recruitment was used as a random effect. For cohorts (RS-III-2, ALSPAC, CHS_EA and CHS_AA) that did not have coffee and tea consumptions measured at the same time as DNA methylation, additional time difference adjustment was introduced in the cohort specific adjustments. The findings from model 2 were considered as a primary results, as it is the most conservative model.

Mendelian Randomization (MR) study

We implemented a two-sample Mendelian randomization (MR) approach to evaluate the potential causal effect of coffee consumption on the identified CpGs, investigating whether the DNA methylation changes are a consequence of coffee consumption (**Figure S3**). We used 50 independent SNPs as instrumental variables (IVs) of coffee consumption, units of cups of coffee per day (including drinkers and non-coffee drinkers) (**Table S2**) [20, 21]. In addition, we assessed the potential causal association between coffee-consumption related CpG and cardiometabolic traits including type 2 diabetes, body mass index, waist-hip ratio, lipid traits (HDL-C, LDL-C, total cholesterol, triglycerides) and coronary heart disease (CHD). For each CpG, we chose instrumental variables for DNA methylation levels based on methylation quantitative trait loci (cis-meQTL) obtained from FHS cohort (N=4170) [22]. The IVs were selected from independent cis-meQTL SNPs pruned by LD $r2 < 0.01$. Genetic association data of cardiometabolic traits were obtained from publicly available GWAs namely DIAGRAM Consortium [23], GIANT consortium [24], ENGAGE consortium [25], and UK-Biobank+ CARDIoGRAMplusC4D consortium [26].

Two methods were used to explore causality. First, a weighted genetic risk score (GRS) was constructed for coffee consumption by multiplying the number of effect alleles at each locus by the corresponding reported β coefficient from the GWAS and then summing the products. The total score was then divided by the average effect size multiplied by 100 to rescale the scores and standardize them to a range between 0 and 100. The other MR approaches were performed using the summary statistics for genetic association of the selected SNPs with coffee consumption and with the CpG site of interest. The causal

effect estimate was obtained through inverse variance weighting (IVW). We further used two sensitivity analyses, the weighted median and MR-Egger, to investigate potential effect of pleiotropic variants on the estimates. The effect sizes and standard errors for SNPs-CpG were obtained from meta-analyzing GWAS summary statistics from the RS and FHS (n=5,371) [22]. We used MR-PRESSO (Mendelian randomization pleitropy residual sum and outlier) to identify horizontal pleiotropic outliers in multi-instrument summary-level MR testing (https://github.com/rondolab/MR-PRESSO) [27]. All MR methods for multiple genetic instruments were conducted using "MendelianRandomization", a statistical package running under R [28].

Funding and Acknowledgements

ALSPAC: The UK Medical Research Council and Wellcome (Grant ref: 217065/Z/19/Z) and the University of Bristol provide core support for ALSPAC. This publication is the work of the authors and Srikant Ambatipudi will serve as guarantor for the contents of this paper. We are extremely grateful to all the families who took part in this study, the midwives for their help in recruiting them, and the whole ALSPAC team, which includes interviewers, computer and laboratory technicians, clerical workers, research scientists, volunteers, managers, receptionists and nurses. CR is funded by the MRC Integrative Epidemiology Unit (MC_UU_12013_2)

Airwave: The Airwave Health Monitoring Study was funded by the Home Office (grant number 780-TETRA, 2003-2018) and is currently funded by the Medical Research Council and Economic & Social Research Council (MR/R023484/1) with additional support from the National Institute for Health Research (NIHR) Biomedical Research Centre. The Airwave Study uses the computing resources of the UK MEDical BIOinformatics partnership (UK MED-BIO supported by the Medical Research Council (MR/L01632X/1). The authors would like to acknowledge Dr. Maryam Karimi in Imperial College for the QC work she performed for the DNA methylation data of Airwave Study. PE and JH acknowledge support from the UK Dementia Research Institute (UK DRI) at Imperial College London funded by the Medical Research Council, Alzheimer's Society and Alzheimer's Research UK. PE is associate director of Health Data Research UK London at Imperial College London which is funded by a consortium of sponsors led by the Medical Research Council.

ARIC: The Atherosclerosis Risk in Communities study has been funded in whole or in part with federal funds from the National Heart, Lung, and Blood Institute, National Institutes of Health, Department of Health and Human Services (contract numbers HHSN268201700001I, HHSN268201700002I, HHSN268201700003I, HHSN268201700004I and HHSN268201700005I). Funding was also supported by 5RC2HL102419 and R01NS087541. The authors thank the staff and participants of the ARIC study for their important contributions.

CHS: The CHS research was supported by NHLBI contracts HHSN268201200036C, HHSN268200800007C, HHSN268201800001C, N01HC55222, N01HC85079, N01HC85080, N01HC85081, N01HC85082, N01HC85083, N01HC85086; and NHLBI grants U01HL080295, R01HL087652, R01HL105756, R01HL103612, R01HL120393, R01HL085251, and U01HL130114 with additional contribution from the National Institute of Neurological Disorders and Stroke (NINDS). Additional support was provided through R01AG023629 from the National Institute on Aging (NIA). A full list of principal CHS investigators and institutions can be found at CHS-NHLBI.org.

The provision of genotyping data was supported in part by the National Center for Advancing Translational Sciences, CTSI grant UL1TR001881, and the National Institute of Diabetes and Digestive and Kidney Disease Diabetes Research Center (DRC) grant DK063491 to the Southern California Diabetes Endocrinology Research Center.

The content is solely the responsibility of the authors and does not necessarily represent the official views of the National Institutes of Health.

EPIC Study (Epic_Italy and Epic_IARC): This work was supported by grants from the Institut National du Cancer (INCa, France) to I Romieu and Z Herceg and the European Commission (EC) Seventh Framework Programme (FP7) Translational Cancer Research (TRANSCAN) Framework to Z Herceg. Z Herceg was also supported by the Fondation ARC pour la Recherche sur le Cancer (France) and the EC FP7 EurocanPlatform: A European Platform for Translational Cancer Research (grant number: 260791). The work reported in this paper was undertaken during the tenure of a Postdoctoral Fellowship (to S Ambatipudi) from the International Agency for Research on Cancer, partially supported by the EC FP7 Marie Curie Actions – People – Co-funding of regional, national and international programmes (COFUND). European prospective Investigation into Cancer and Nutrition (EPIC) is funded through World Health Organization (WHO) and Europe Against Cancer Program of the European Commission.

ESTHER study: The ESTHER study was funded by grants from the Saarland state Ministry for Social Affairs, Health, Women and Family Affairs (Saarbrücken, Germany), the Baden-Württemberg state Ministry of Science, Research and Arts (Stuttgart, Germany), the Federal Ministry of Education and Research (Berlin, Germany) and the Federal Ministry of Family Affairs, Senior Citizens, Women and Youth (Berlin, Germany) and the Saarland State Ministry of Social Affairs, Health, Women and Family (Saarbrücken, Germany). We thank Volker Herrmann and Christa Stegmaier for their help in setting up the ESTHER cohort and Melanie Bewerunge-Hudler and Matthias Schick for their help in creating the methylation database.

FHS: The Framingham Heart Study is supported by National Instituted of Health Contract N01-HC-25195; HHSN268201500001I.

KORA: The KORA study was initiated and financed by the Helmholtz Zentrum München – German Research Center for Environmental Health, which is funded by the German Federal Ministry of Education and Research (BMBF) and by the State of Bavaria. This work was supported by the German Federal Ministry of Education and Research (BMBF) within the framework of the EU Joint Programming Initiative 'A Healthy Diet for a Healthy Life' (DIMENSION grant number 01EA1902A).

Lifelines: The work was supported by the Dutch Digestive Disease Foundation (MLDS WO11-30 to CW), the European Research Council Advanced Grant (ERC-671274 to CW), the Netherlands Organization for Scientific Research (NWO-VENI grant 916-10135 to LF and NWO VIDI grant 917-14374 to LF) and the European Union's Seventh Framework Programme (EU FP7) TANDEM project (HEALTH-F3-2012-305279 to CW).

Rotterdam Study: The generation and management of the Illumina 450K methylation array data (EWAS data) for the Rotterdam Study was executed by the Human Genotyping Facility of the Genetic Laboratory of the Department of Internal Medicine, Erasmus MC, the Netherlands. The EWAS data was funded by the Genetic Laboratory of the Department of Internal Medicine, Erasmus MC, and by the Netherlands Organization for Scientific Research (NWO; project number 184021007) and made available as a Rainbow Project (RP3; BIOS) of the Biobanking and Biomolecular Research Infrastructure Netherlands (BBMRI-NL). We would like to extend our gratitude to all the participants

providing the data, practitioners collecting the data and experts cleaning and preparing the data, thus making this study feasible for the epidemiological analysis. We would also like to acknowledge Dr. Athina Vidaki from Department of Genetic Identification, Erasmus MC, for her help in interpretation of the results in this particular project.

TwinsUK: The TwinsUK study was funded by the Welcome Trust; European Community's Seventh Framework Programme (FP7/2007-2013). The study also receives support from the National Institute for Health Research (NIHR)-funded BioResource, Clinical Research Facility and Biomedical Research Centre based at Guy's and St Thomas' NHS Foundation Trust in partnership with King's College London. This project also received support from the JPI ERA-HDHL DIMENSION project and UK Biological Sciences Research Council (BBSRC, BB/S020845/1 and BB/T019980/1 to JTB).

Supplementary References

1. Fraser A, Macdonald-Wallis C, Tilling K, Boyd A, Golding J, Smith GD, Henderson J, Macleod J, Molloy L, Ness A, et al: Cohort Profile: The Avon Longitudinal Study of Parents and Children: ALSPAC mothers cohort. *International Journal of Epidemiology* 2013, 42:97-110.
2. Fraser A, Macdonald-Wallis C, Tilling K, Boyd A, Golding J, Davey Smith G, Henderson J, Macleod J, Molloy L, Ness A: Cohort profile: the Avon Longitudinal Study of Parents and Children: ALSPAC mothers cohort. *International journal of epidemiology* 2013, 42:97-110.
3. Yang TO, Crowe F, Cairns BJ, Reeves GK, Beral V: Tea and coffee and risk of endometrial cancer: cohort study and meta-analysis. *American Journal of Clinical Nutrition* 2015, 101:570-578.
4. McGuire S: Institute of Medicine. 2014. Caffeine in Food and Dietary Supplements: Examining Safety-Workshop Summary. Washington, DC: The National Academies Press, 2014. *Adv Nutr* 2014, 5:585-586.
5. Elliott P, Vergnaud AC, Singh D, Neasham D, Spear J, Heard A: The Airwave Health Monitoring Study of police officers and staff in Great Britain: rationale, design and methods. *Environ Res* 2014, 134:280-285.
6. The Atherosclerosis Risk in Communities (ARIC) Study: design and objectives. The ARIC investigators. *Am J Epidemiol* 1989, 129:687-702.
7. Kumanyika SK, Tell GS, Shemanski L, Martel J, Chinchilli VM: Dietary assessment using a picture-sort approach. *American Journal of Clinical Nutrition* 1997, 65:1123-1129.
8. Gonzalez CA, Riboli E: Diet and cancer prevention: Contributions from the European Prospective Investigation into Cancer and Nutrition (EPIC) study. *European Journal of Cancer* 2010, 46:2555-2562.
9. Zhang Y, Wilson R, Heiss J, Breitling LP, Saum KU, Schottker B, Holleczek B, Waldenberger M, Peters A, Brenner H: DNA methylation signatures in peripheral blood strongly predict all-cause mortality. *Nat Commun* 2017, 8:14617.
10. Gao X, Mons U, Zhang Y, Breitling LP, Brenner H: DNA methylation changes in response to active smoking exposure are associated with leukocyte telomere length among older adults. *Eur J Epidemiol* 2016, 31:1231-1241.
11. Low M, Stegmaier C, Ziegler H, Rothenbacher D, Brenner H: Epidemiological investigations of the chances of preventing, recognizing early and optimally treating chronic diseases in an elderly population (ESTHER study). *Deutsche Medizinische Wochenschrift* 2004, 129:2643-2647.
12. Kannel WB, Feinleib M, Mcnamara PM, Garrison RJ, Castelli WP: Investigation of Coronary Heart-Disease in Families - Framingham Offspring Study. *American Journal of Epidemiology* 1979, 110:281-290.
13. Ikram MA, Brusselle GGO, Murad SD, van Duijn CM, Franco OH, Goedegebure A, Klaver CCW, Nijsten TEC, Peeters RP, Stricker BH, et al: The Rotterdam Study: 2018 update on objectives, design and main results. *Eur J Epidemiol* 2017, 32:807-850.
14. Voortman T, Kiefte-de Jong JC, Ikram MA, Stricker BH, van Rooij FJA, Lahousse L, Tiemeier H, Brusselle GG, Franco OH, Schoufour JD: Adherence to the 2015 Dutch dietary guidelines and risk of non-communicable diseases and mortality in the Rotterdam Study. *European journal of epidemiology* 2017, 32:993-1005.
15. Verdi S, Abbasian G, Bowyer RCE, Lachance G, Yarand D, Christofidou P, Mangino M, Menni C, Bell JT, Falchi M: TwinsUK: The UK Adult Twin Registry Update. *Twin Research and Human Genetics* 2019:1-7.
16. Kurushima Y, Tsai P-C, Castillo-Fernandez J, Alves AC, Moustafa JSE-S, Le Roy C, Spector TD, Ide M, Hughes FJ, Small KS: Epigenetic findings in periodontitis in UK twins: a cross-sectional study. *Clinical epigenetics* 2019, 11:27.
17. Mulligan A, Luben R, Bhaniani B et al. A new tool for converting food frequency questionnaire data into nutrient and food group values: FETA research methods and availability. *BMJ Open* 2014;4:e004503

18. Sandoval J, Heyn H, Moran S, Serra-Musach J, Pujana MA, Bibikova M, Esteller M: Validation of a DNA methylation microarray for 450,000 CpG sites in the human genome. *Epigenetics* 2011, 6:692-702.
19. Houseman EA, Accomando WP, Koestler DC, Christensen BC, Marsit CJ, Nelson HH, Wiencke JK, Kelsey KT: DNA methylation arrays as surrogate measures of cell mixture distribution. *BMC Bioinformatics* 2012, 13:86.
20. Kwok MK, Leung GM, Schooling CM: Habitual coffee consumption and risk of type 2 diabetes, ischemic heart disease, depression and Alzheimer's disease: a Mendelian randomization study. *Scientific reports* 2016, 6:36500.
21. Kennedy OJ, Pirastu N, Poole R, Fallowfield JA, Hayes PC, Grzeszkowiak EJ, Taal MW, Wilson JF, Parkes J, Roderick PJ: Coffee Consumption and Kidney Function: A Mendelian Randomization Study. *American Journal of Kidney Diseases* 2019.
22. Huan T, Joehanes R, Song C, Peng F, Guo Y, Mendelson M, Yao C, Liu C, Ma J, Richard M: Genome-wide identification of DNA methylation QTLs in whole blood highlights pathways for cardiovascular disease. *Nature communications* 2019, 10:1-14.
23. Mahajan A, Taliun D, Thurner M, Robertson NR, Torres JM, Rayner NW, Payne AJ, Steinthorsdottir V, Scott RA, Grarup N: Fine-mapping type 2 diabetes loci to single-variant resolution using high-density imputation and islet-specific epigenome maps. *Nature genetics* 2018, 50:1505-1513.
24. Pulit SL, Stoneman C, Morris AP, Wood AR, Glastonbury CA, Tyrrell J, Yengo L, Ferreira T, Marouli E, Ji Y: Meta-analysis of genome-wide association studies for body fat distribution in 694 649 individuals of European ancestry. *Human molecular genetics* 2019, 28:166-174.
25. Surakka I, Horikoshi M, Mägi R, Sarin A-P, Mahajan A, Lagou V, Marullo L, Ferreira T, Miraglio B, Timonen S: The impact of low-frequency and rare variants on lipid levels. *Nature genetics* 2015, 47:589.
26. van der Harst P, Verweij N: Identification of 64 novel genetic loci provides an expanded view on the genetic architecture of coronary artery disease. *Circulation research* 2018, 122:433-443.
27. Verbanck M, Chen C-y, Neale B, Do R: Detection of widespread horizontal pleiotropy in causal relationships inferred from Mendelian randomization between complex traits and diseases. *Nature genetics* 2018, 50:693-698.
28. Yavorska OO, Burgess S: MendelianRandomization: an R package for performing Mendelian randomization analyses using summarized data. *International journal of epidemiology* 2017, 46:1734-1739.

Supplementary Figures 1-14:

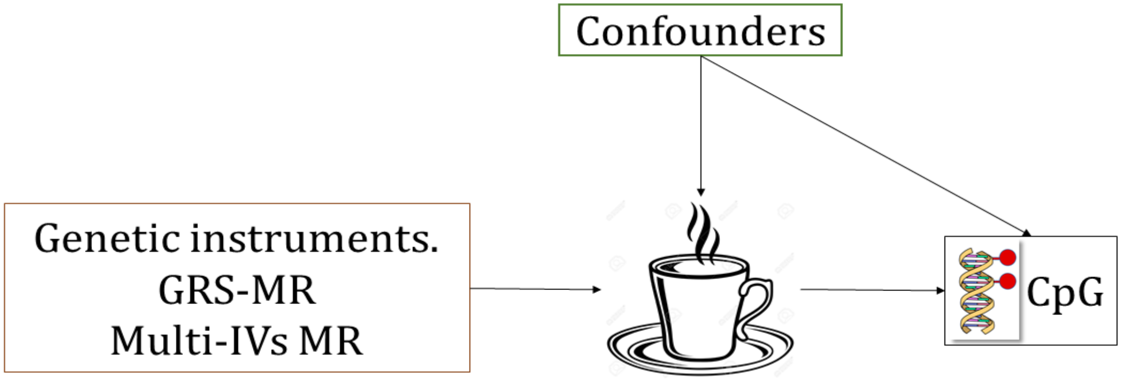

Supplementary Figure 1. Graphical representation of the causal association between coffee consumption and DNA methylation. GRS, Genetic risk score; IVs, Instrumental variables; CpG, DNA methylation site; MR, Mendelian randomization.

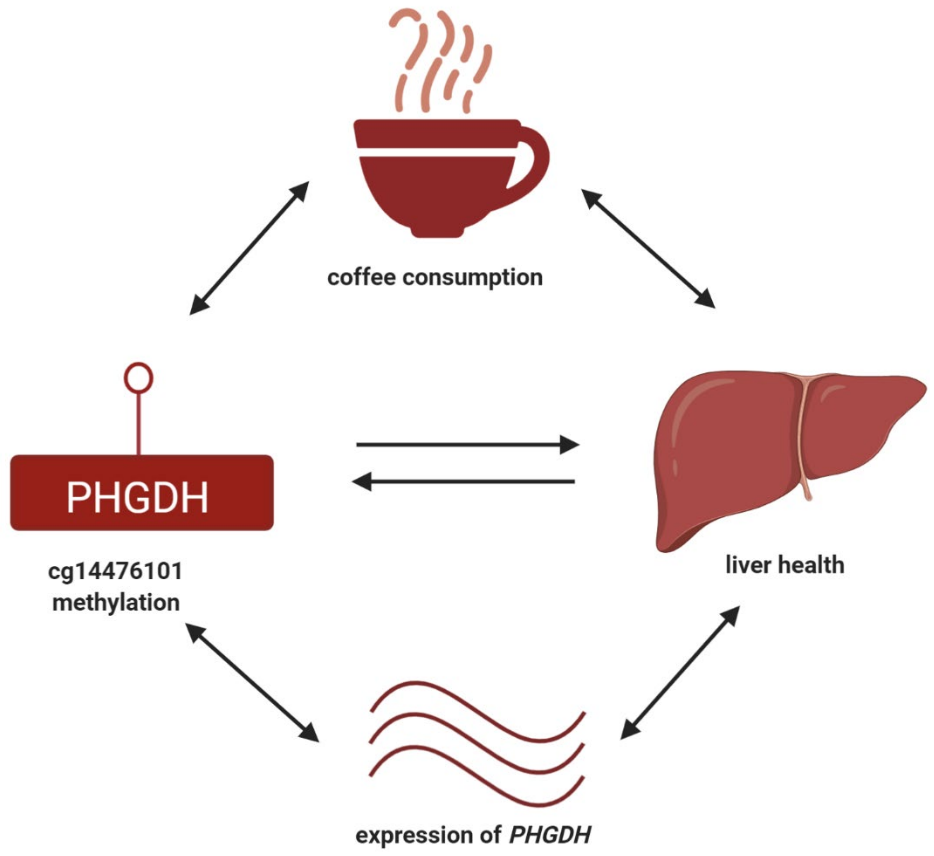

Supplementary Figure 2. **Schematic overview of the analysis for cg14476101 annotated to the *PHGDH* gene in relation to liver diseases.**

Created with BioRender.com.

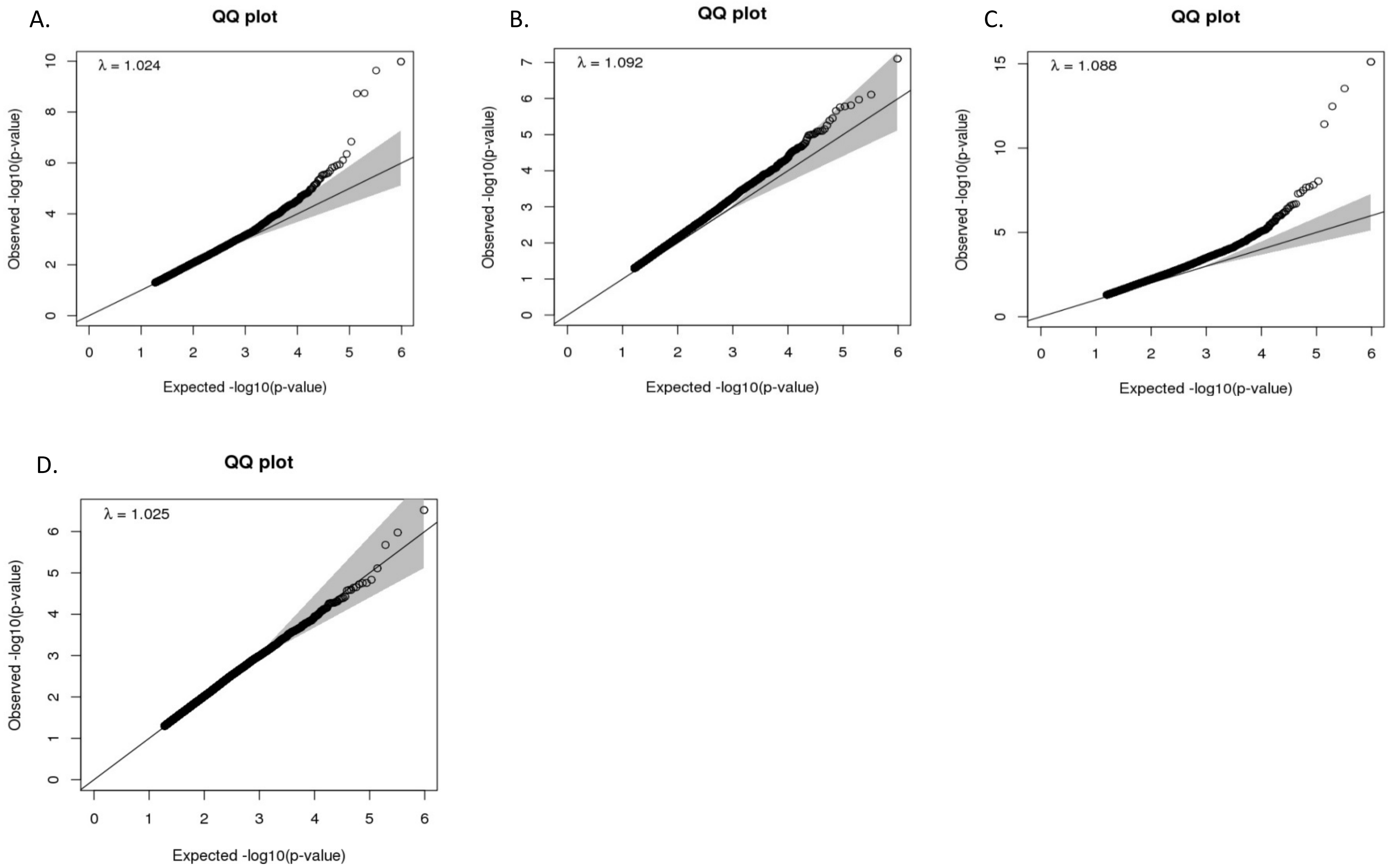

Supplementary Figure 3. **Computed Quantile-Quantile (QQ) plots with the corresponding inflation factor (lambda values) for model 2.** The x-axis corresponds to the expected –log10 p-value under the null hypothesis, while the y-axis indicates observed –log10 p-value. Plot (A) depicts epigenome–wide association study discovery phase (n=9,612) for coffee consumption and plot (B) depicts replication phase (n=6,177) with coffee consumption. Plots (C) and (D) correspond to the overall sample size meta-analysis on coffee consumption (n=15,789) and tea consumption (n=15,069), respectively.

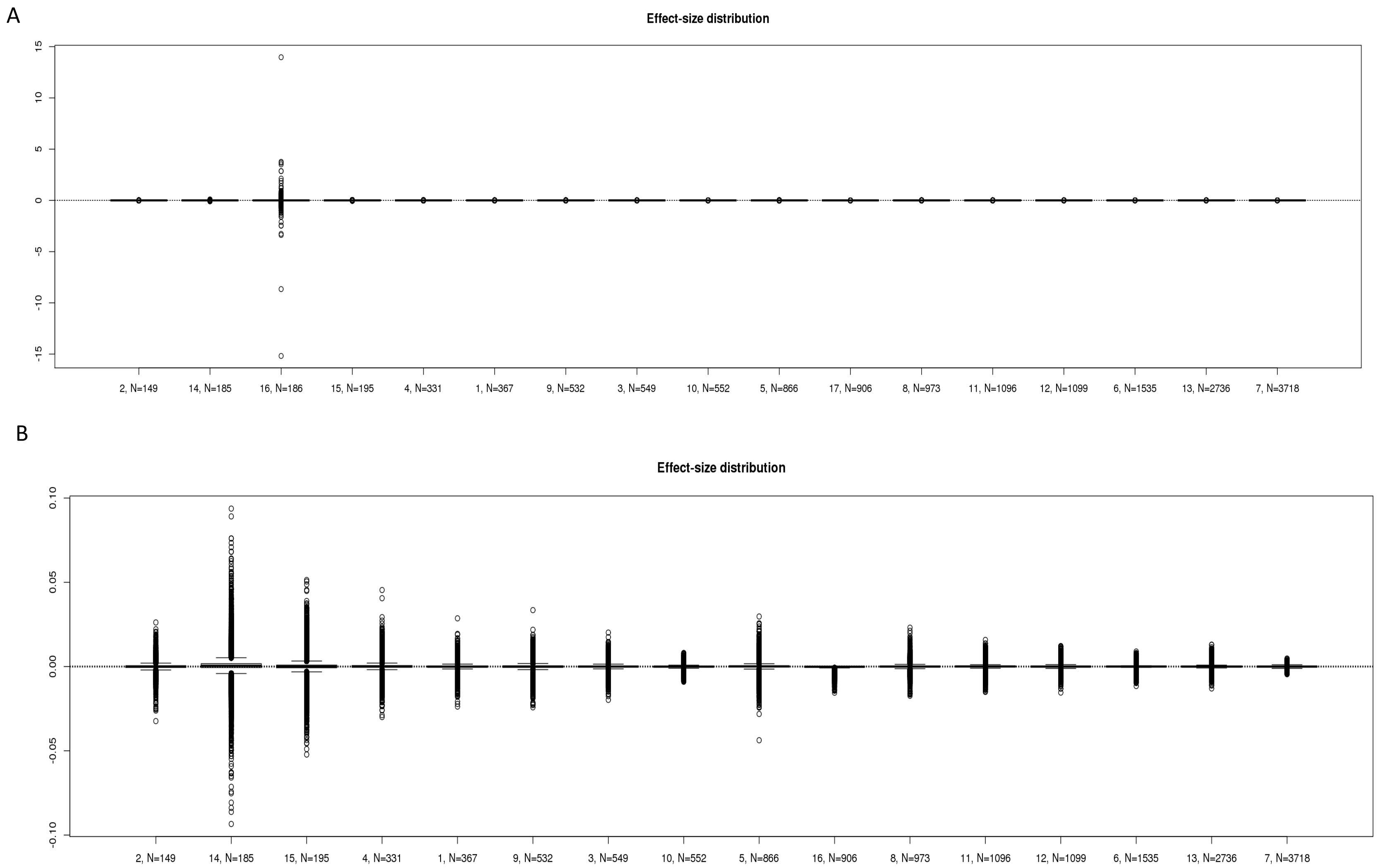

A
Effect-size distribution
15
10
5
0
-5
-10
-15
2, N=149
14, N=185
16, N=186
15, N=195
4, N=331
1, N=367
9, N=532
3, N=549
10, N=552
5, N=866
17, N=906
8, N=973
11, N=1096
12, N=1099
6, N=1535
13, N=2736
7, N=3718
B
Effect-size distribution
0.10
0.05
0.00
-0.05
-0.10
2, N=149
14, N=185
15, N=195
4, N=331
1, N=367
9, N=532
3, N=549
10, N=552
5, N=866
16, N=906
8, N=973
11, N=1096
12, N=1099
6, N=1535
13, N=2736
7, N=3718

C

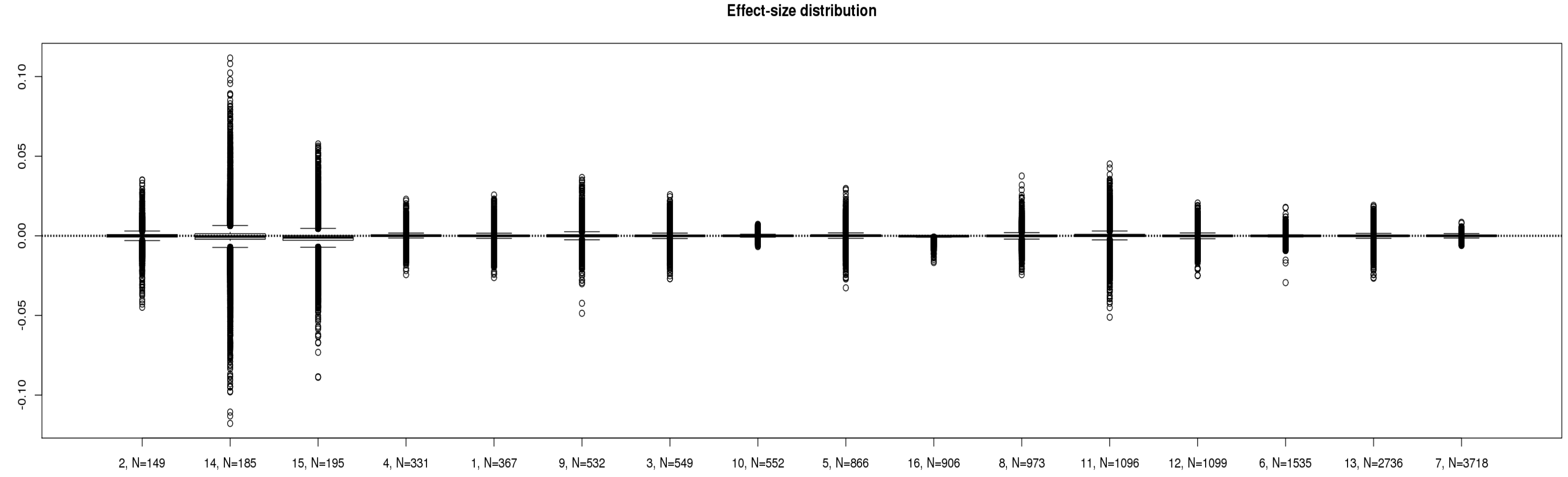

Supplementary Figure 4. **Effect-size distribution plots for coffee and tea consumption**. Plot (A) depicts the effect-size distribution plot with Lifelines cohort, while plot (B) depicts the effect-size distribution plot without Lifelines cohort with coffee consumption. There is a notably wider spread of effect sizes in the Lifelines cohort than expected based on sample size, which could indicate different units of measurement or analysis model. Plot (C) depicts the effect-size distribution plot with all participating cohorts for tea consumption.

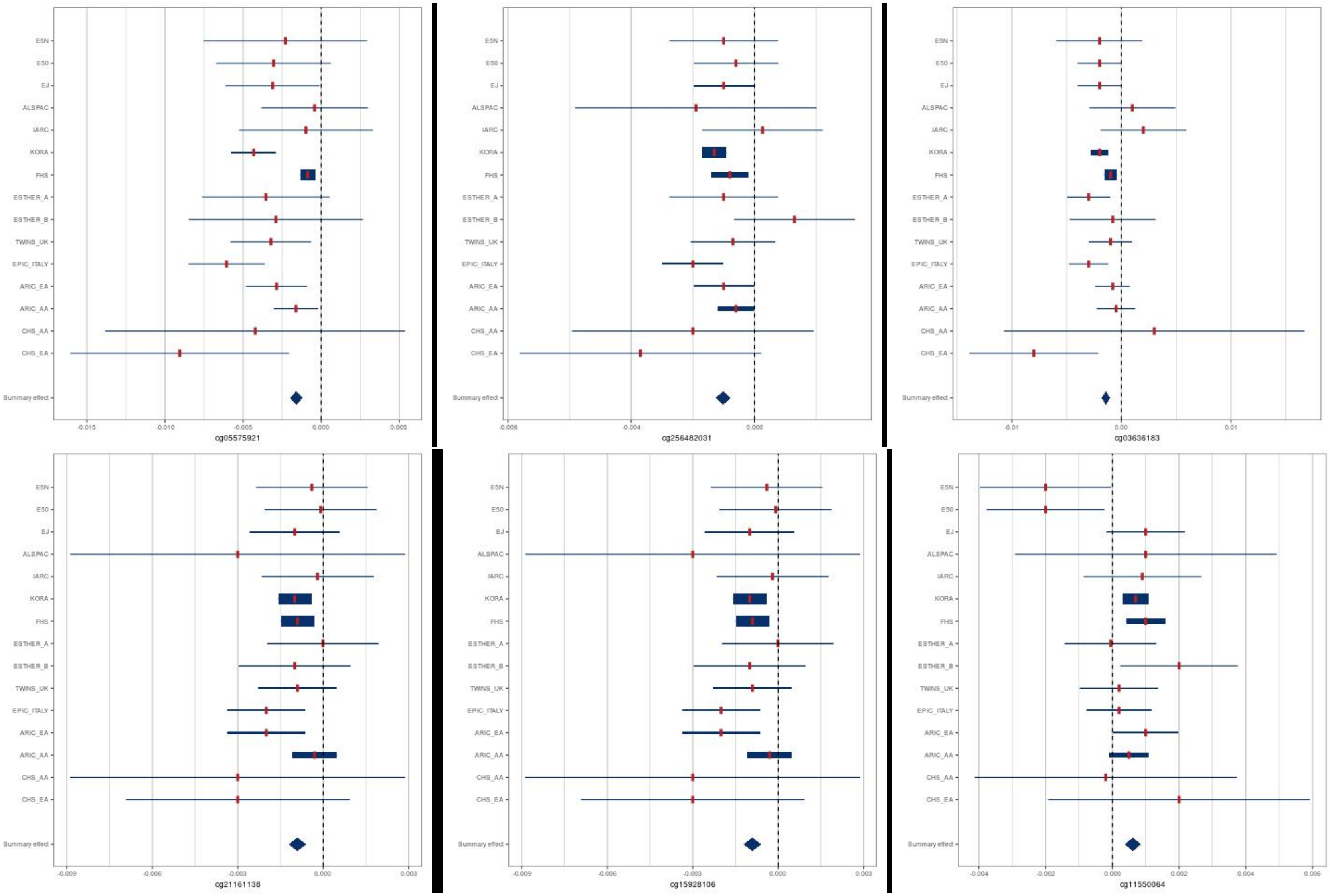
E5N
E50
EJ
ALSPAC
IARC
KORA
FHS
ESTHER_A
ESTHER_B
TWINS_UK
EPIC_ITALY
ARIC_EA
ARIC_AA
CHS_AA
CHS_EA
Summary effect
-0.015
-0.010
-0.005
0.000
0.005
cg05575921
-0.008
-0.004
0.000
cg256482031
-0.01
0.00
0.01
cg03636183
-0.009
-0.006
-0.003
0.000
0.003
cg21161138
-0.009
-0.006
-0.003
0.000
0.003
cg15928106
-0.004
-0.002
0.000
0.002
0.004
0.006
cg11550064

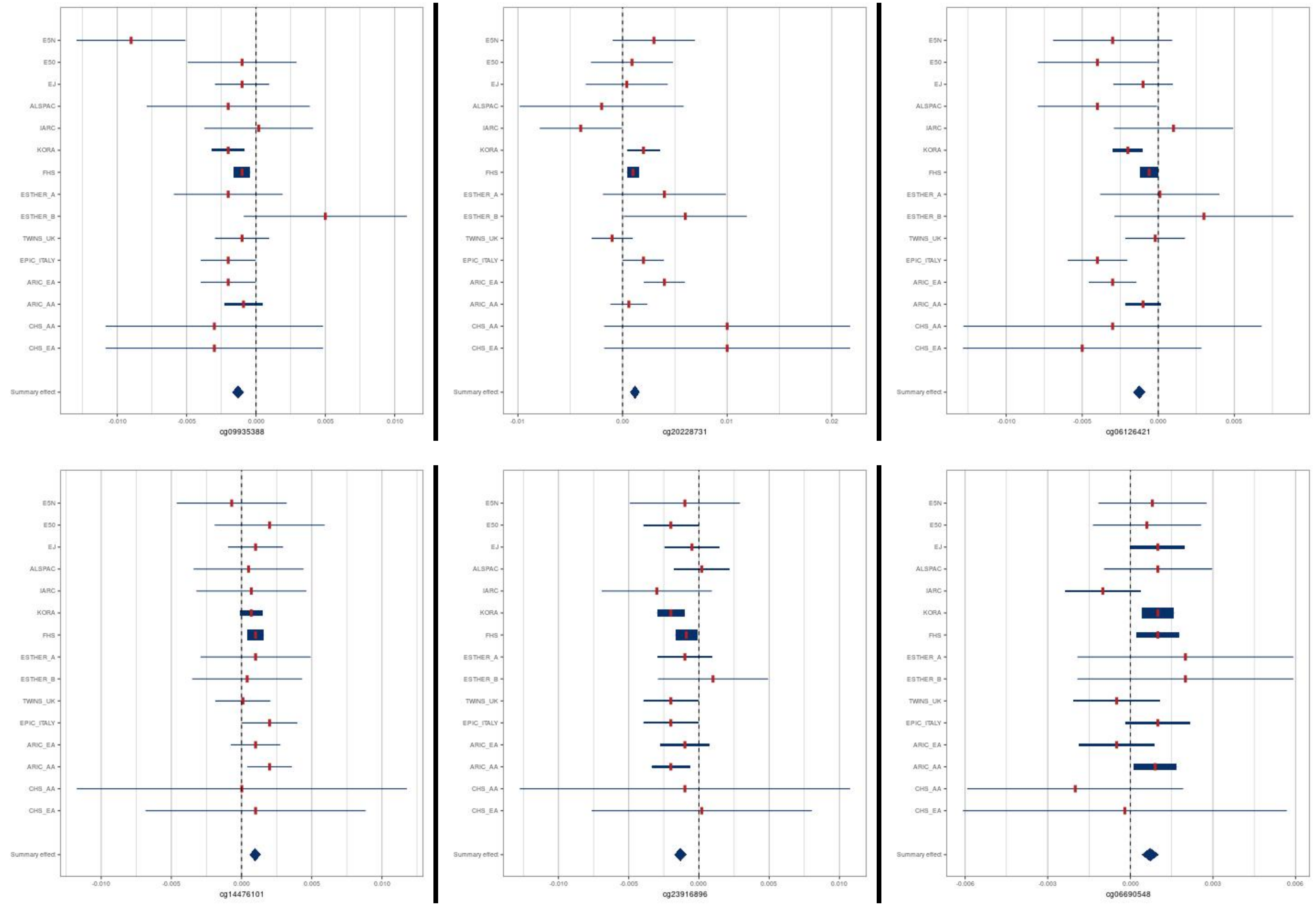

Supplementary Figure 5. **Forest plots depicting direction of effects for the 11 significant and 1 suggestive coffee associated CpGs.** Forest plots computed for the top 15 CpGs associated with coffee consumption in β effects and standard errors (SE) (n=15,789). Effect estimates of individual studies with their 95%

confidence intervals are illustrated, where line width is proportional to the weight assigned to the study in the meta-analysis. As expected, the cohorts with smaller sample size have a wider SE. Rectangular error bars are used to display confidence intervals, as well as the relative meta-analytic weight (height of the error bar) of each study.

A.

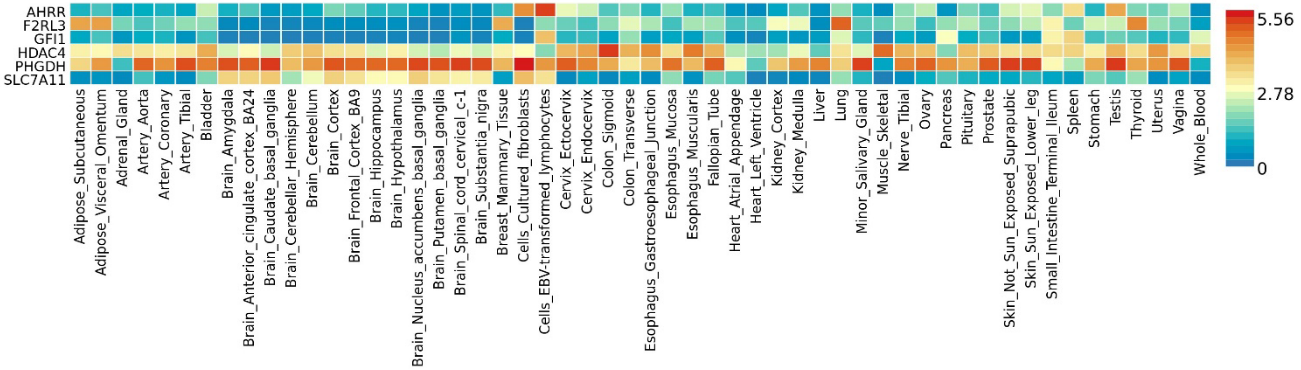

B.

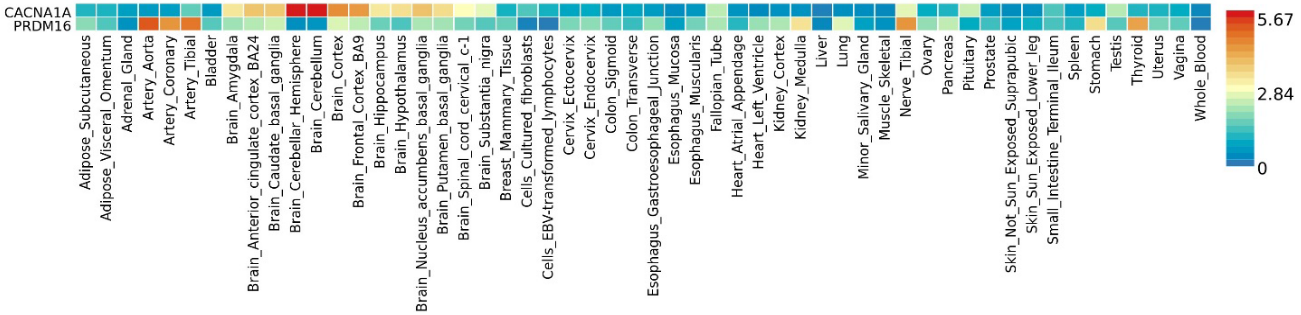

Supplementary Figure 6. **Heatmap depicting an average expression of genes annotated to the coffee-associated CpGs (A) and tea-suggestive associated CpGs (B) across 53 c tissues provided by GTEx**. Colors indicate the average expression value (log2 transformed Reads Per Kilobase per Million per tissue per gene, winsorization 50). Darker red color indicates higher expression of the gene, while darker blue represents lower expression level. This Figure is downloaded from the official GTEx through FUMA GWAS (www.funa.ctglab.nl). GTEx=Genotype-Tissue Expression. FUMA GWAS= Functional Mapping and Annotation of Genome-wide Association Studies

A.

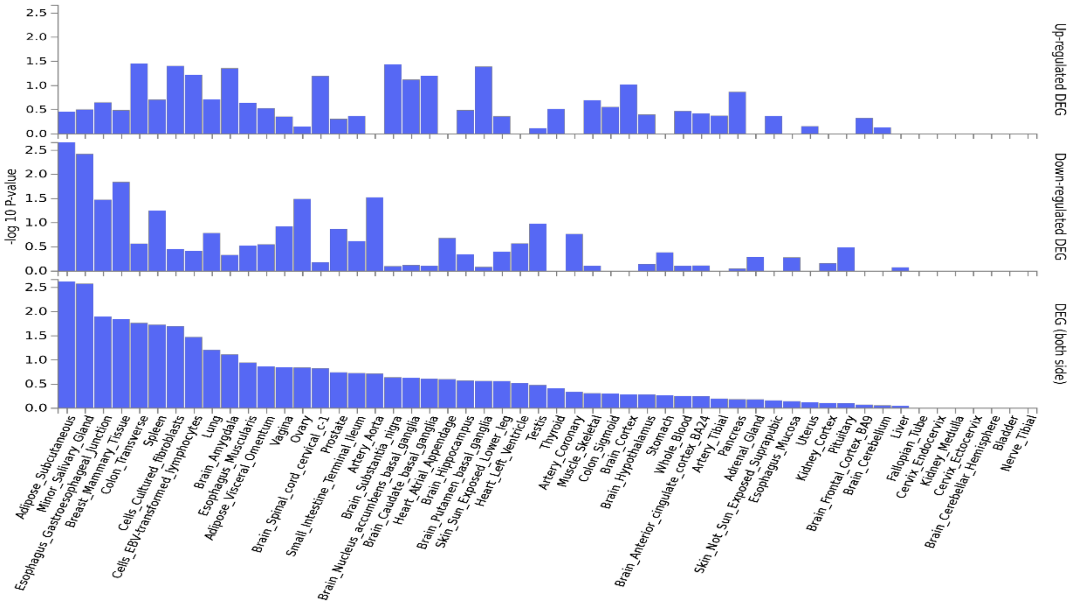

B.

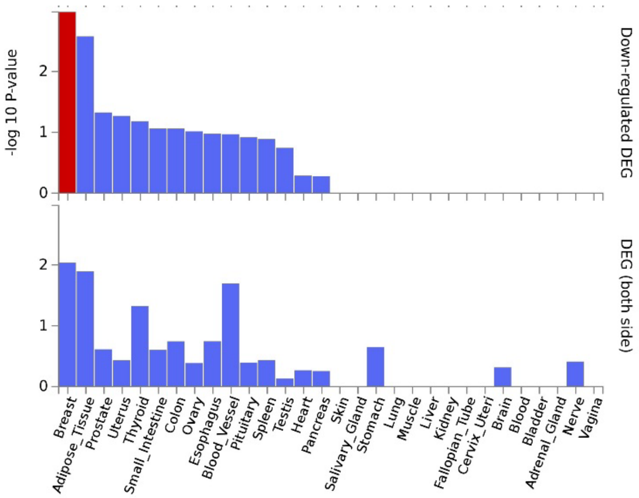

Supplementary Figure 7. Tissue expression of the genes annotated to CpGs associated with coffee (A) and tea consumption (B). Specific tissue types generated by GTEx in FUMA, where red indicates significantly enriched DEG sets (Pbon < 0.05).

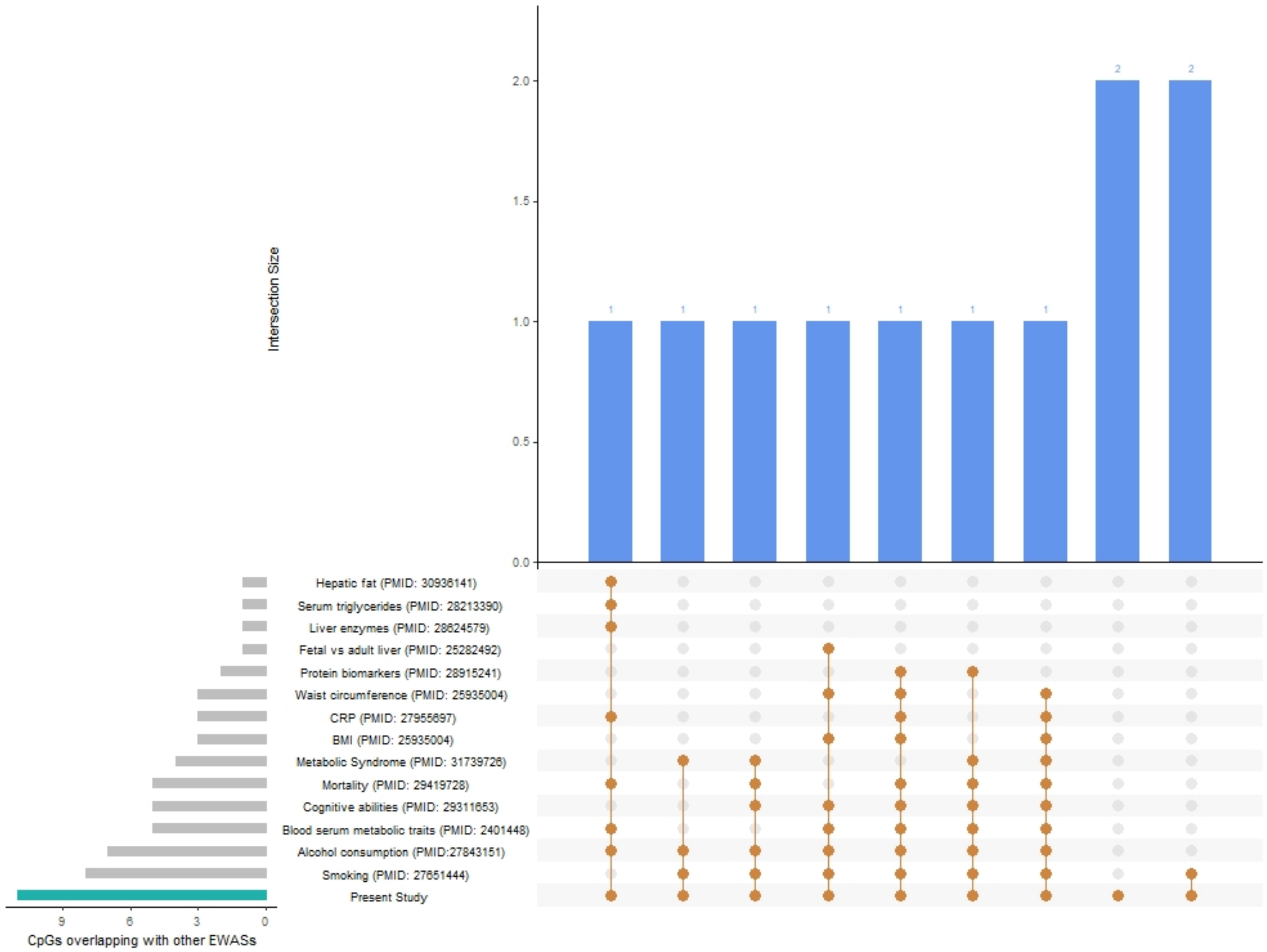

Supplementary Figure 8. **The UpSet plot depicts the number of overlapping CpGs identified in several previous EWAS.** The horizontal bar plot represents the number of coffee associated CpGs from our study and number of overlapping CpGs from several previous EWASs. The vertical bar plot depicts the number of CpGs in each set, denoted by the connected circles below the histogram.

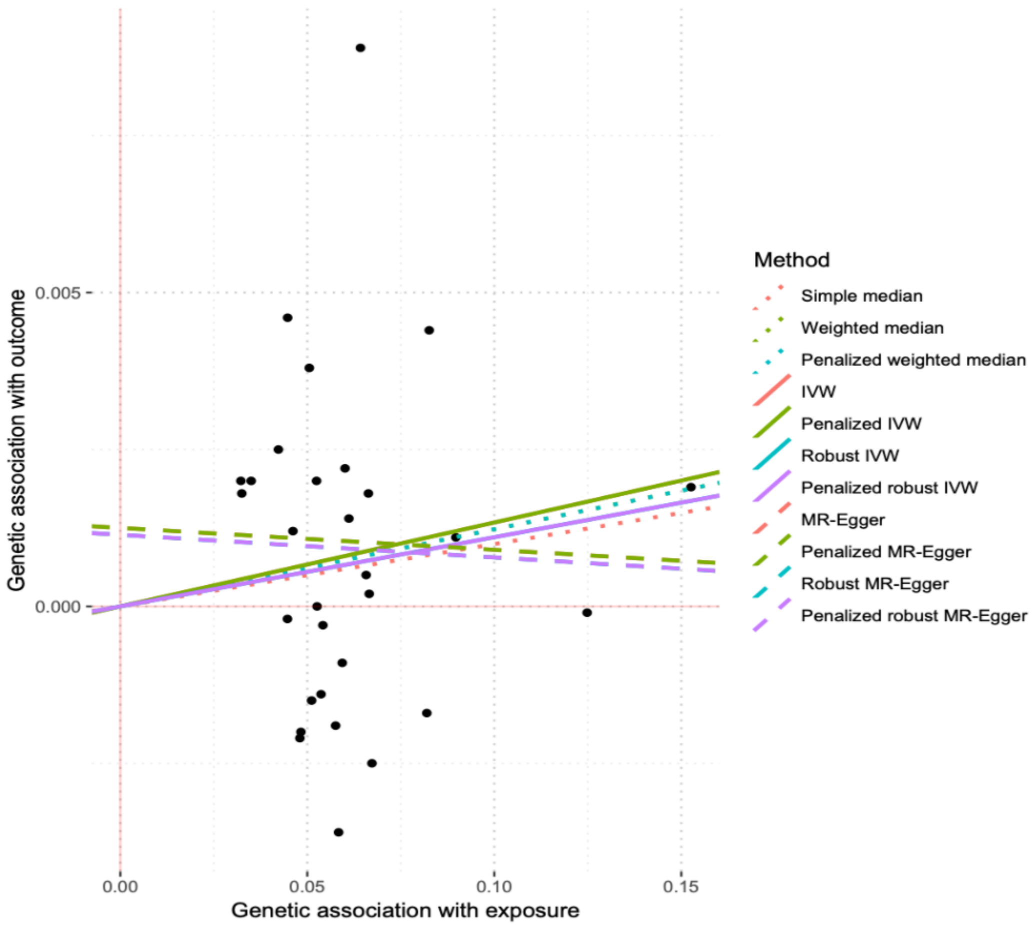

Supplementary Figure 9. Two-sample Mendelian randomization plot regarding coffee consumption (GWAS n=357454, 50 SNPs) and cg14476101. The Y axis represents the genetic association estimates with outcome (cg14476101). The X axis represents the genetic association estimate with exposure (coffee consumption). Lines depict the causal estimates from the different Mendelian randomization methods.

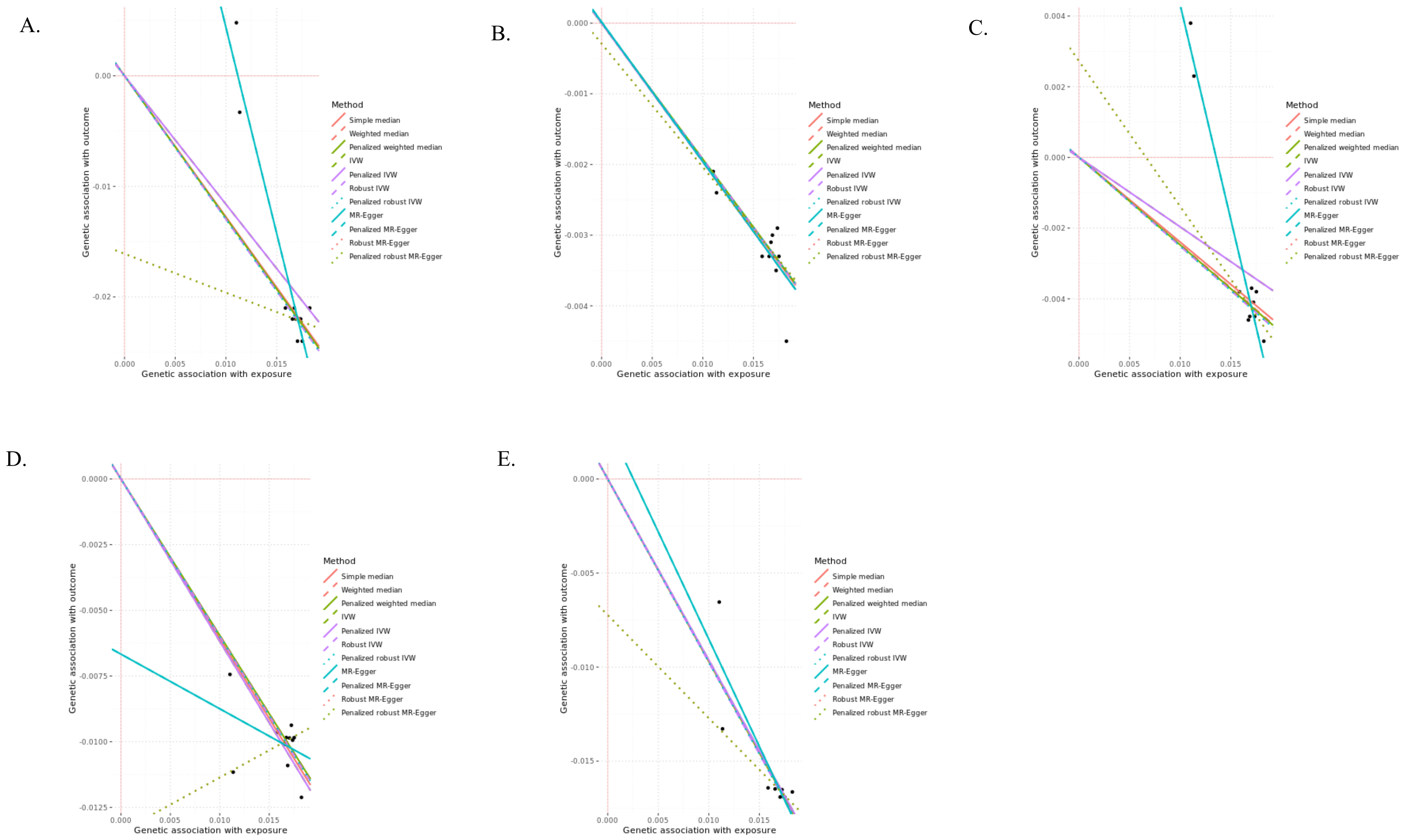

Supplementary Figure 10. **Two-sample Mendelian randomization plots regarding cg01940273 (n=4170, 11 SNPs) and type 2 diabetes (n=898130) (A), body mass index (n=694649) (B), waist-hip ratio (n=694649) (C), LDL-C (n=62166) (D) and total cholesterol (n=62166) (E).** The Y axis represents the genetic association estimates with outcome. The X axis represents the genetic association estimate with exposure (cg01940273). Lines depict the causal estimates from the different Mendelian randomization methods

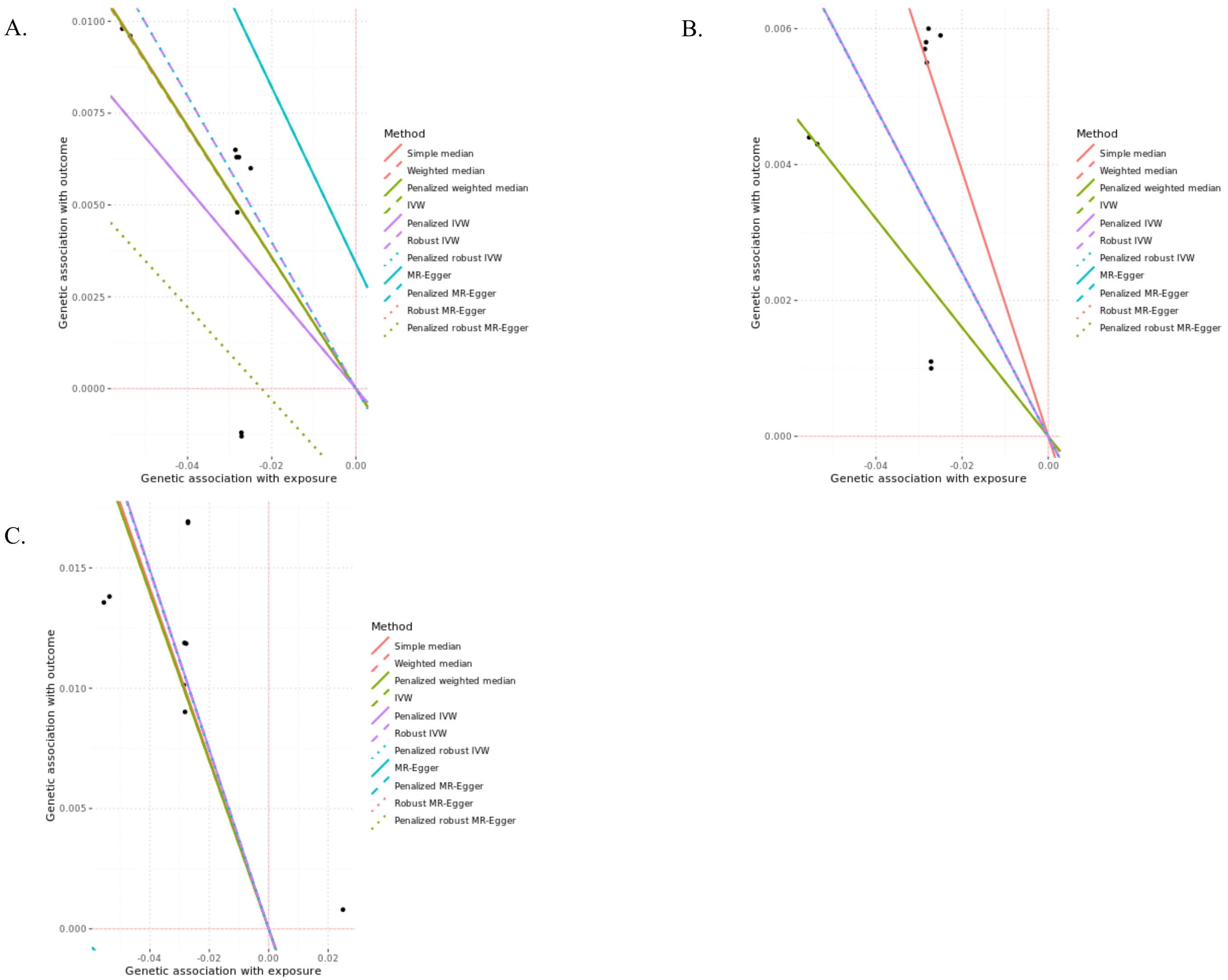

Supplementary Figure 11. Two-sample Mendelian randomization plots regarding cg05575921 (n=4170, 9 SNPs) and body mass index (n=694649) (A), waist-hip ratio (n=694649) (B), and HDL-C (n=62166) (C). The Y axis represents the genetic association estimates with outcome. The X axis represents the genetic association estimate with exposure (cg05575921). Lines depict the causal estimates from the different Mendelian randomization methods

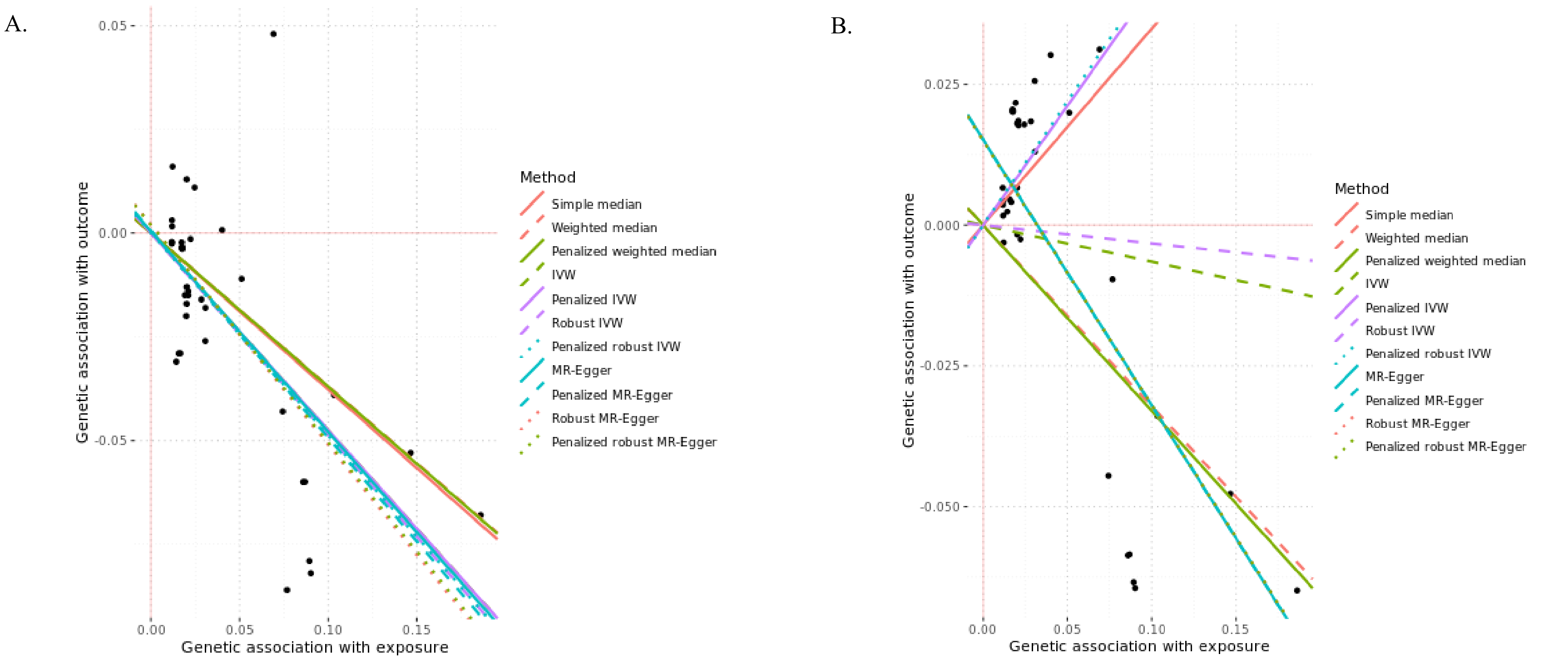

Supplementary Figure 12. **Two-sample Mendelian randomization plots regarding cg09935388 (n=4170, 37 SNPs) and type 2 diabetes (n=898130) (A), and HDL-C (n=62166) (B).** The Y axis represents the genetic association estimates with outcome. The X axis represents the genetic association estimate with exposure (cg09935388). Lines depict the causal estimates from the different Mendelian randomization methods

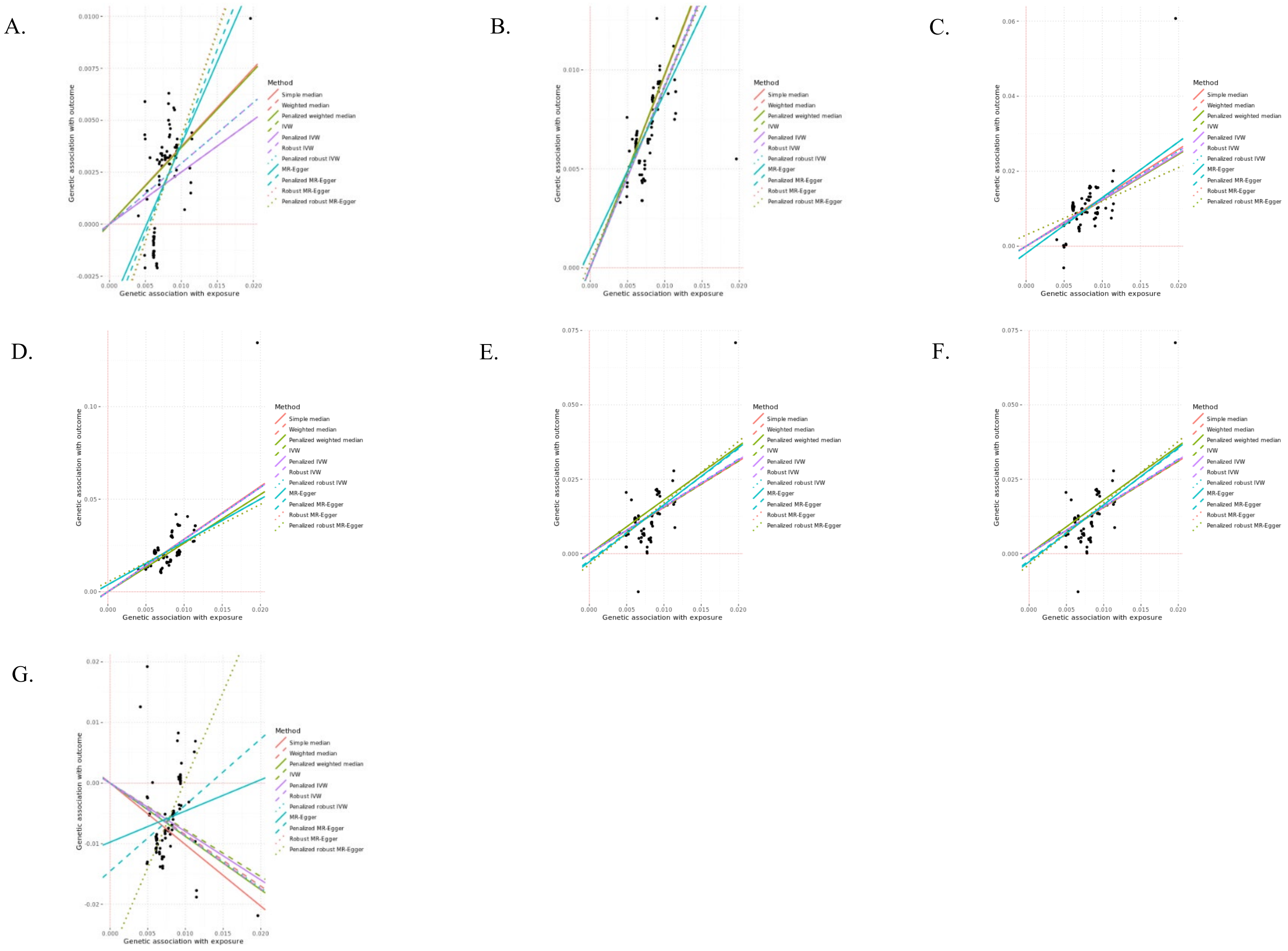

Supplementary Figure 13. Two-sample Mendelian randomization plots regarding cg11550064 (n=4170, 85 SNPs) with body mass index (n=694649) (A), waist-hip ratio (n=694649) (B), HDL-C (n=62166) (C), LDL-C (n=62166) (D), total cholesterol (n=62166) (E), triglycerides (n=62166) (F) and coronary

heart disease (n=547261) (G). The Y axis represents the genetic association estimates with outcome. The X axis represents the genetic association estimate with exposure (cg11550064). Lines depict the causal estimates from the different Mendelian randomization methods

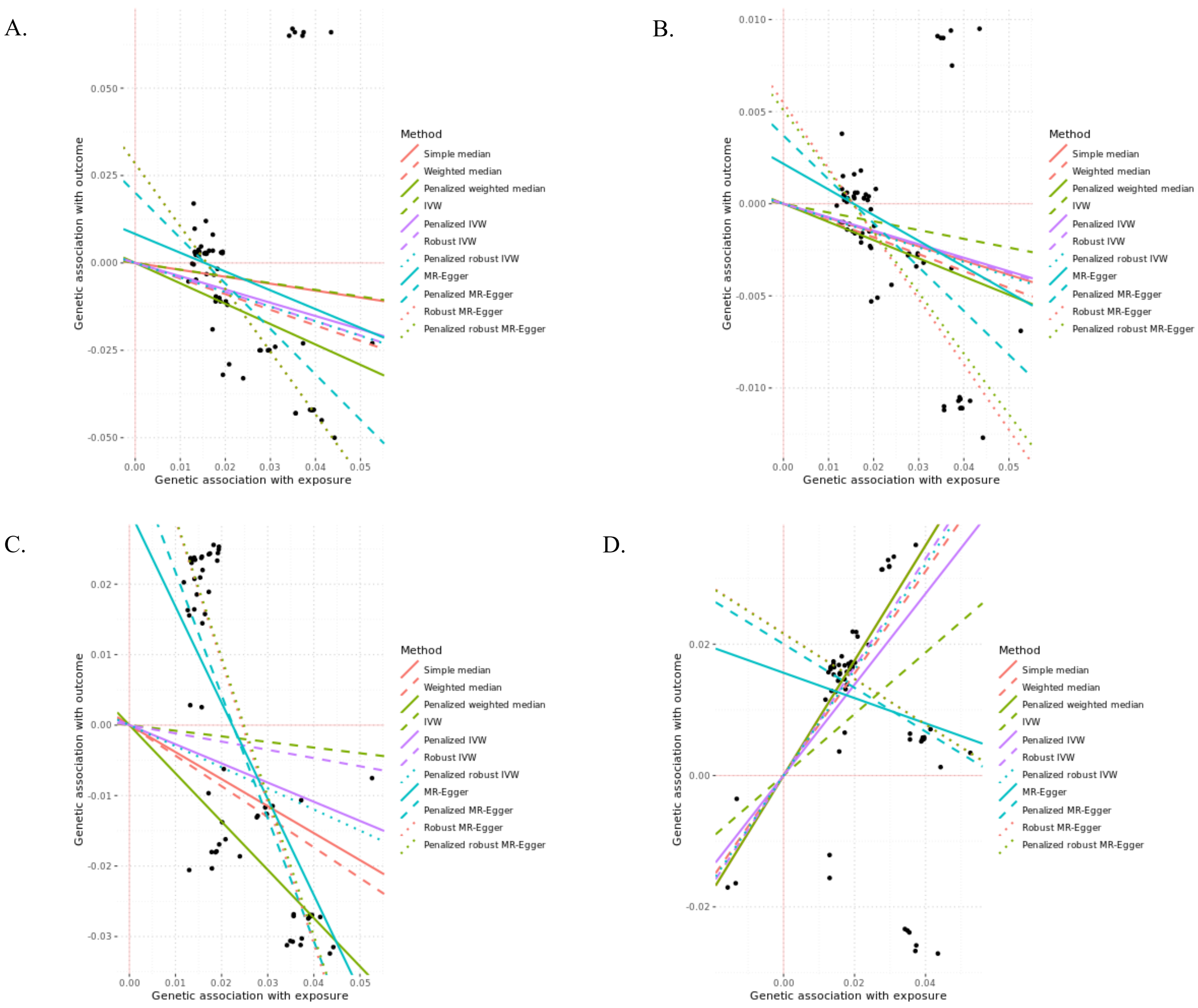

Supplementary Figure 14. Two-sample Mendelian randomization plots regarding cg23916896 (n=4170, 66 SNPs) with type 2 diabetes (n=898130) (A), body mass index (n=694649) (B), HDL-C (n=62166) (C) and total cholesterol (n=62166) (D). The Y axis represents the genetic association estimates with

outcome. The X axis represents the genetic association estimate with exposure (cg23916896). Lines depict the causal estimates from the different Mendelian randomization methods

Supplementary Table 1. DNA methylation methods in different cohorts

Cohort	Methylation assay	IDAT extraction	Background correction	Detection P value cut-off	Sample call rate treshold	Nbeads filter	Normalization
RS-III-2	Illumina 450K	Beadstudio	separate colors	NA	95%	NA	DASEN
RS-II-3	Illumina 450K	Beadstudio	separate colors	NA	95%	NA	DASEN
RS-III-1	Illumina 450K	Custom script	separate colors	0.01	NA	3	SWAN
ALSPAC	Illumina 450K	Meffil	NA	0.01	95%	3	Functional Normalization
KORA	Illumina 450K	Custom script	separate colors	0.01	95%	3	CPACOR
FHS	Illumina 450K	Minfi	separate colors	0.01	99%	NA	DASEN
ESTHER_A	Illumina 450K	GenomeStudio	separate colors	0.01	95%	NA	Illumina
ESTHER_B	Illumina 450K	GenomeStudio	separate colors	0.01	95%	NA	Illumina
TwinsUK	Illumina 450K	Beadstudio	spearate colors	0.000001	95%	3	DASEN
Airwave	Illumina 850K	NA	NA	NA	0.95	NA	DASEN
Lifelines	Illumina 450K	Beadstudio	separate colors	NA	95%	NA	DASEN
ARIC_EA	Illumina 450K	Genome Studio	separate colors	0.01	95%	NA	BMIQ
ARIC_AA	Illumina 450K	Genome Studio	separate colors	0.01	95%	NA	BMIQ
CHS_EA	Illumina 450K	Genome Studio	NA	0.01	95%	NA	SWAN
CHS_AA	Illumina 450K	Genome Studio	NA	0.01	95%	NA	SWAN
EPIC_Italy	Illumina 450K	Genome Studio	separate colors	0.01	95%	3	BMIQ
EPIC_IARC	Illumina 450K	Genome Studio	separate colors	0.01	95%	3	BMIQ

RS=Rotterdam Study. ALSPAC=The Avon Longitudinal Study of Parents and Children. KORA= Cooperative Health Research in the Augsburg Region Study.

Supplementary Table 2. Genetic instruments for coffee consumption

SNP	Locus	Nearest gene	EA	NEA	β	SD
rs1260326	2p24	*GCKR*	C	T	0.040	0.010
rs1481012	4q22	*ABCG2*	A	G	0.060	0.010
rs17685	7q11.23	*POR*	A	G	0.070	0.010
rs2470893	15q24	*CYP1A1*	T	C	0.120	0.010
rs2472297	15q24	*CYP1A2*	T	C	0.140	0.010
rs4410790	7p21	*AHR*	C	T	0.100	0.010
rs6265	11p13	*BDNF*	C	T	0.040	0.010
rs7800944	7q11.23	*MLXIPL*	C	T	0.050	0.010
rs9902453	17q11.2	*EFCAB5*	G	A	0.030	0.010
rs10851873	15q24.1	*ARID3B*	C	T	0.058	0.010
rs11742322	5p15.31	*LINC02142*	C	T	-0.035	0.006
rs11855112	15q24.1	*TBC1D21*	C	T	0.054	0.008
rs12539460	7p21.1	*LOC101927630*	T	C	-0.045	0.006
rs12901093	15q24.1	*CCDC33*	G	A	0.032	0.006
rs12917120	15q24.2	*PPCDC*	C	T	0.059	0.006
rs13233604	7p21.1	*LOC101927630*	A	T	-0.052	0.008
rs13397165	chr2	-	A	G	-0.046	0.007
rs1463237	12p13.2	*PRH1*	T	C	-0.044	0.007
rs16903275	5q14.3	*LINC00461*	A	C	0.044	0.008
rs17427548	15q24.2	*UBE2Q2*	G	A	0.066	0.012
rs17644994	7p21.1	*LOC101927609*	T	G	-0.067	0.011
rs17645813	7p21.1	*KCCAT333*	A	G	-0.090	0.010
rs17702298	7p21.1	*LOC105375171*	A	G	0.082	0.012
rs17706320	7p21.1	*LOC101927630*	C	T	-0.053	0.006
rs17817964	16q12.2	*FTO*	T	C	0.041	0.006
rs1877723	4p16.3	*ADD1*	T	C	-0.035	0.006
rs2231142	4q22.1	*ABCG2*	T	G	-0.061	0.009
rs2305668	15q24.1	*SCAMP2*	G	T	-0.048	0.009
rs2504706	chr6	-	C	T	-0.037	0.006
rs2647003	chr6	-	G	T	-0.032	0.006
rs351242	15q24.1	*STRA6*	A	G	-0.060	0.006
rs3810291	19q13.32	*ZC3H4*	A	G	0.036	0.006
rs4077582	15q24.1	*CYP11A1*	T	C	0.051	0.006
rs4128436	15q24.1	*EDC3*	T	C	-0.066	0.010
rs4665386	2p23.2	*LINC01460*	A	C	0.045	0.008
rs476828	18q21.32	*MC4R*	C	T	0.047	0.006
rs4886593	15q24.1	*CCDC33*	A	T	-0.054	0.007
rs589500	chr1	-	T	C	0.040	0.007
rs6062678	20q13.33	*PCMTD2*	T	G	0.038	0.006
rs6461314	7p21.1	*KCCAT333*	G	A	0.064	0.009
rs660550	6p21.33	*SLC44A4*	A	C	-0.036	0.005
rs6792502	3p21.31	*CACNA2D2*	A	C	-0.046	0.008
rs7224815	17p11.2	*TOM1L2*	T	A	-0.032	0.006
rs7251570	chr19	-	G	A	0.037	0.006
rs7458455	7p21.1	*LOC101927609*	T	G	0.048	0.008
rs7791070	7:17361403	*AHR*	C	T	-0.083	0.006
rs799166	chr7	-	G	C	0.058	0.008

Supplementary Table 3. Quantitative real-time polymerase chain reaction (qRT-PCR) primer sequences

shRNA sequence 1 for PHGDH	CCGGCAGACTTCACTGGTGTC AGATCTCGAGATCTGACACCAGTGAAGTCTGTTTTT
shRNA sequence 2 for PHGDH	CCGGCGCAGAACTCACTTGTGG AATCTCGAGATTCCACAAGTGAGTTCTGCGTTTTT
Target sequence 1	CAGACTTCACTGGT GTCAGAT
Target sequence 2	CGCAGAACTCACTTGTGGAAT

Supplementary Table 4. Inverse-variance random effects meta-analysis of EWAS with coffee consumption

CpG	CHR	Position	Gene ID	metaZ	P-value	I^2
cg05575921	5	373378	AHRR	-4.600441	4.28E-06	0.7
cg15928106	7	130646078	FLJ43663	2.554978	0.010639	0.52
cg11550064	2	240148191	HDAC4	3.184801	0.001455	0.47
cg20228731	7	130646051	FLJ43663	2.56858	0.010231	0.48
cg06126421	6	30720080	NA	-3.81366	1.38E-04	0.43

Model adjusted for sex, age, smoking WBCs, technical covariates, BMI and alcohol. P value treshold was set at P < 0.01 (5/0.05), based on 5 CpGs that showed nominal evidence of heterogeneity from the main analysis. CpG= DNA methylation site; CHR = chromosome.

Supplementary Table 5. Previous GWA and EWA studies on coffee and tea consumptions

Coffee consumption			
PMID	Study	Year	Genes
21876539	GWAS	2011	*CYP1A1, CYP1A2, ULK3, CPLX3, NCALD, LAMB4, NRCAM*
27702941	GWAS	2016	*AC073332.1, AHR, AC019117.4, AC012435.2, AC012435.3, UBL7-AS1, AC012435.3, ARID3B, CLK3, CYP1A1, CYP1A2, NUMBL, CYP2F2P, AC008537.1, AC008537.1, CYP2A6, CYP2G1P, CYP2A7P2, CYP2B6, CYP2B7P, GCKR*
27561104	GWAS	2016	*PDSS2, RPS24P12*
25288136	GWAS	2014	*GCKR, ABCG2, AHR, AC073332.1, POR, AC103796.1, CYP1A1, CYP1A2, EFCAB5, MLXIPL, OPCML, DPY19L4P2, AC004911.1*
21357676	GWAS	2011	*CYP1A2, CYP1A1, AHR, AC073332.1*
29367735	GWAS	2018	*HECTD4, AC016553.1, LINC02220, AL355538.2, ATP1A1, AHR, AC073332.1*
31345160	GWAS	2019	*SORCS2, AC073332.1, AHR, CUX2*
31959922	GWAS	2020	*MCL1, ENSA, GCKR, AGR3, AHR, ALDH2, CYP1A2, CSK, ADORA2A-AS1, AC073332.1, ABCG2, AL589740.1, MLXIPL, POR, APOA5, AP006216.2, BDNF, BDNF-AS, CYP2G1P, CYP2A7P2, AC019117.2, AC019117.4, AC019117.3, AC019117.2, MDH2*
31837886	GWAS and MR	2019	*DENND1B, GCKR, ADD1, ABCG2, SLC44A4, MTCO3P1, AL662789.1, AHR, AC073332.1, AC019117.4, AC019117.3, AC019117.2, MLXIPL, POR, AC018943.1, INSYN1-AS1, STRA6, CCDC33, AC090826.1, AC090826.2, CLK3, EDC3, CYP1A1, CYP1A2, PPCDC, PPCDC, AC090771.1, RNU4-17P*
28535255	EWAS	2017	-
28198392	EWAS	2017	*ALPPL2* (cg21566642) (* in model unadjusted for smoking)
Tea consumption			
31959922	GWAS (*green tea)	2020	*ALDH2*
28535255	EWAS	2017	*DNAJC16* (cg18192808), *TTC17* (cg14055589) (* in women only)

Supplementary Table 6. Association of lead SNPs of CpG-meQTLS with coffee consumption

CpG	No. meQTLS	lead SNP	β	SE	P-value
cg05575921	116	rs2721011	-0.0009	0.006	0.9
cg25648203	138	rs2466287	0.002	0.008	0.8
cg23916896	523	rs11133981	0.001	0.007	0.8
cg21161138*	-	-	-	-	-
cg03636183	41	rs2227383	0.009	0.005	0.08
cg15928106	11	rs17738934	0.004	0.005	0.5
cg11550064	116	rs291336	0.007	0.008	0.4
cg09935388	43	rs114297139	-0.01	0.01	0.5
cg20228731*	-	-	-	-	-
cg06126421*	-	-	-	-	-
cg14476101	222	rs11583993	0.01	0.008	0.2
cg06690548*	-	-	-	-	-

* no significant meQTLs found
meQTLS evaluated in 4,170 subjects from Framingham Heart Study
P-value <0.006 (Bonferroni adjusted 0.05/8 SNPs)

Supplementary Table 7. Meta-analysis EWAS on coffee consumption in all samples and only samples with the same time point of DNA methylation and coffee consumption

				Overall sample (n=15,789)		Only samples with same time point (n=15,181)	
CpG	CHR	Position	Gene ID	β	P-value	β	P-value
cg05575921	5	373378	AHRR	-0.0016	2.17E-15	-0.0015	8.81E-15
cg25648203	5	395444	AHRR	-0.001	7.31E-14	-0.001	1.14E-12
cg03636183	19	17000585	F2RL3	-0.0014	1.15E-12	-0.0014	4.33E-12
cg21161138	5	399360	AHRR	-0.0011	6.66E-12	-0.0011	1.85E-11
cg15928106	7	130646078	FLJ43663	0.0015	1.59E-08	0.0014	8.91E-08
cg11550064	2	240148191	HDAC4	0.0007	2.11E-08	0.0007	1.02E-08
cg09935388	1	92947588	GFI1	-0.0012	2.32E-08	-0.0011	3.90E-07
cg20228731	7	130646051	FLJ43663	0.0015	3.87E-08	0.0014	1.23E-07
cg06126421	6	30720080	NA	-0.0011	4.50E-08	-0.0011	3.12E-07
cg14476101	1	120255992	PHGDH	0.0011	4.71E-08	0.0012	3.39E-08
cg23916896	5	368804	AHRR	-0.0013	4.76E-08	-0.0013	3.54E-08

P-value $<1.1\times10^{-7}$ (Bonferroni adjusted 0.05/450,000)

Supplementary Table 8. Transethnic meta-analysis EWAS on coffee consumption

CpG	CHR	Position	Gene ID	Discovery EA (N=13,146)		Replication AA (N=2,921)	
				β	P-value	β	P-value
cg05575921	5	373378	AHRR	-0.0016	3.62E-14	-0.0017	0.018
cg25648203	5	395444	AHRR	-0.0011	4.01E-13	-0.0007	0.028
cg03636183	19	17000585	F2RL3	-0.0014	8.01E-13	-0.0005	0.625
cg21161138	5	399360	AHRR	-0.0012	1.39E-12	-0.0004	0.374
cg13711966	2	42071660		0.0009	1.15E-08	-0.0004	0.334
cg15928106	7	130646078	FLJ43663	0.0015	2.67E-08	0.0011	0.292
cg20228731	7	130646051	FLJ43663	0.0015	5.72E-08	0.001	0.312
cg09935388	1	92947588	GFI1	-0.0013	5.81E-08	-0.001	0.168
cg11550064	2	240148191	HDAC4	0.0007	6.34E-08	0.0005	0.11
CpG	CHR	Position	Gene ID	Discovery AA (N=2,921)		Replication EA (N=13,146)	
				β	P-value	β	P-value
cg05822739	4	3504980		-0.0015	1.08E-07	-0.0003	0.068
cg10055139	11	111797552	C11orf52	0.0016	2.71E-07	0	0.249
cg09067818	2	43864246	PLEKHH2	0.0008	7.75E-07	0	0.685

In the Discovery phase, P-value trehold was considered at P-value $<1.1\times10^{-7}$ (Bonferroni adjusted 0.05/450,000). While for replication phase, it corresponds to number of significantly associated CpG sites from disovery phase divided by 0.05 (Bonferroni adjusted).

Supplementary Table 9. Sensitivity analyses meta-analysis EWAS on coffee consumption

Stratification analyses				
Strata	CpG	β	SE	P-value
men (n= 2,129)				
men	cg09935388	-0.0024	0.001	0.01146
men	cg14476101	0.0007	0.0009	0.4125
men	cg11550064	0.0006	0.0005	0.2279
men	cg06690548	0.0022	0.0006	0.0006946
men	cg21161138	-0.001	0.0005	0.07111
men	cg23916896	-0.0009	0.0008	0.2416
men	cg25648203	-0.0007	0.0005	0.1542
men	cg06126421	0	0.0009	0.9641
men	cg15928106	0.0036	0.0013	0.004243
men	cg20228731	0.0035	0.0013	0.007902
men	cg05575921	-0.0016	0.0011	0.1468
men	cg03636183	-0.0017	0.0008	0.03241
women (n=2,654)				
women	cg09935388	-0.0009	0.0009	0.3235
women	cg14476101	0.0012	0.0009	0.1791
women	cg11550064	0.0015	0.0005	0.001842
women	cg06690548	0.0006	0.0006	0.3064
women	cg21161138	-0.0007	0.0005	0.1514
women	cg23916896	-0.0007	0.0008	0.3338
women	cg25648203	-0.0007	0.0005	0.1419
women	cg06126421	-0.0012	0.0009	0.1908
women	cg15928106	0.0045	0.0012	0.0001862
women	cg20228731	0.004	0.0013	0.001515
women	cg05575921	-0.0019	0.0009	0.03743
women	cg03636183	-0.0014	0.0008	0.06588
low-drinkers (n=1,650)				
low-drinkers	cg09935388	-0.0018	0.0055	0.739
low-drinkers	cg14476101	-0.0112	0.0052	0.0327
low-drinkers	cg11550064	0.0032	0.0028	0.259
low-drinkers	cg06690548	0.0016	0.0049	0.7373
low-drinkers	cg21161138	0.0034	0.003	0.2542
low-drinkers	cg23916896	0.0018	0.0044	0.6766
low-drinkers	cg25648203	-0.0009	0.0027	0.729
low-drinkers	cg06126421	0.0024	0.0052	0.6471
low-drinkers	cg15928106	0.0078	0.0071	0.2725
low-drinkers	cg20228731	0.0098	0.0076	0.1991
low-drinkers	cg05575921	0.0021	0.0052	0.6848
low-drinkers	cg03636183	0.005	0.0043	0.2472
moderate-drinkers (n=2,728)				
moderate-drinkers	cg09935388	-0.0012	0.0016	0.4687
moderate-drinkers	cg14476101	0.0051	0.0015	0.0005673
moderate-drinkers	cg11550064	0.0001	0.0008	0.8923
moderate-drinkers	cg06690548	0.0039	0.0011	0.0006497
moderate-drinkers	cg21161138	-0.0011	0.0009	0.2193
moderate-drinkers	cg23916896	0.0001	0.0013	0.9401
moderate-drinkers	cg25648203	-0.0002	0.0008	0.8277
moderate-drinkers	cg06126421	0.0015	0.0015	0.3138
moderate-drinkers	cg15928106	0.005	0.0021	0.01836
moderate-drinkers	cg20228731	0.0045	0.0022	0.04242
moderate-drinkers	cg05575921	-0.0008	0.0017	0.6157
moderate-drinkers	cg03636183	0.0002	0.0013	0.8775
high-drinkers (n=501)				
high-drinkers	cg09935388	0.0009	0.0033	0.7835
high-drinkers	cg14476101	0.0035	0.0024	0.1438
high-drinkers	cg11550064	0.0021	0.0019	0.28
high-drinkers	cg06690548	0.0015	0.0012	0.1902
high-drinkers	cg21161138	-0.0019	0.0021	0.3673
high-drinkers	cg23916896	0.0037	0.0027	0.1672
high-drinkers	cg25648203	-0.003	0.0017	0.07798
high-drinkers	cg06126421	0.0035	0.0032	0.2732
high-drinkers	cg15928106	0.0089	0.0042	0.03226
high-drinkers	cg20228731	0.0056	0.0041	0.1687
high-drinkers	cg05575921	-0.0047	0.0048	0.3245

high-drinkers	cg03636183	-0.0003	0.003	0.9275
non-smokers (n=2,027)				
non-smokers	cg09935388	-0.0005	0.0009	0.5925
non-smokers	cg14476101	0.0019	0.0009	0.04408
non-smokers	cg11550064	0.0016	0.0005	0.003203
non-smokers	cg06690548	0.0014	0.0006	0.02222
non-smokers	cg21161138	-0.0008	0.0005	0.1117
non-smokers	cg23916896	-0.0013	0.0009	0.1239
non-smokers	cg25648203	0.0001	0.0005	0.8765
non-smokers	cg06126421	-0.0007	0.0008	0.4021
non-smokers	cg15928106	0.0029	0.0014	0.04236
non-smokers	cg20228731	0.0021	0.0015	0.1579
non-smokers	cg05575921	-0.0004	0.0006	0.4721
non-smokers	cg03636183	-0.0008	0.0006	0.2211
former-smokers (n=2,280)				
former-smokers	cg09935388	-0.0011	0.0008	0.1853
former-smokers	cg14476101	0.0012	0.0008	0.1363
former-smokers	cg11550064	0.0009	0.0005	0.06043
former-smokers	cg06690548	0.0001	0.0005	0.8665
former-smokers	cg21161138	0.0003	0.0005	0.5917
former-smokers	cg23916896	0.0006	0.0007	0.4508
former-smokers	cg25648203	0	0.0004	0.9925
former-smokers	cg06126421	-0.0003	0.0009	0.7194
former-smokers	cg15928106	0.0026	0.0012	0.02926
former-smokers	cg20228731	0.0026	0.0012	0.03102
former-smokers	cg05575921	0.0001	0.0009	0.9287
former-smokers	cg03636183	-0.0006	0.0007	0.3887
current-smokers (n=568)				
current-smokers	cg09935388	-0.0043	0.0021	0.04394
current-smokers	cg14476101	0.0037	0.0015	0.01127
current-smokers	cg11550064	0.0012	0.0009	0.1799
current-smokers	cg06690548	0.0012	0.0011	0.2671
current-smokers	cg21161138	-0.0016	0.0011	0.1437
current-smokers	cg23916896	-0.0018	0.0013	0.1711
current-smokers	cg25648203	-0.0027	0.0011	0.01332
current-smokers	cg06126421	0.0001	0.0019	0.9484
current-smokers	cg15928106	0.0005	0.0021	0.7955
current-smokers	cg20228731	0.0024	0.0023	0.2841
current-smokers	cg05575921	-0.0041	0.0026	0.1241
current-smokers	cg03636183	-0.0033	0.0018	0.07537
**EWAS Coffee adjusted by tea (n=5,172)**				
EWAS.Coffee.TeaAdj	cg09935388	-0.0012	0.0007	0.05929
EWAS.Coffee.TeaAdj	cg14476101	0.0014	0.0006	0.01674
EWAS.Coffee.TeaAdj	cg11550064	0.0009	0.0003	0.006845
EWAS.Coffee.TeaAdj	cg06690548	0.001	0.0004	0.02197
EWAS.Coffee.TeaAdj	cg21161138	-0.0003	0.0004	0.3661
EWAS.Coffee.TeaAdj	cg23916896	-0.0003	0.0005	0.625
EWAS.Coffee.TeaAdj	cg25648203	-0.0006	0.0003	0.0695
EWAS.Coffee.TeaAdj	cg06126421	-0.0004	0.0006	0.5248
EWAS.Coffee.TeaAdj	cg15928106	0.0038	0.0009	0.00001001
EWAS.Coffee.TeaAdj	cg20228731	0.0036	0.0009	0.00006681
EWAS.Coffee.TeaAdj	cg05575921	-0.0012	0.0007	0.1034
EWAS.Coffee.TeaAdj	cg03636183	-0.0012	0.0006	0.03633
**EWAS Tea adjusted by coffee (n=1,454)**				
EWAS.Tea.CoffeeAdj	cg20099906	-4.5E-05	0.0007	0.9
EWAS.Tea.CoffeeAdj	cg05804170	-0.00019	0.0002	0.5

P-value $<4.2\times10^{-3}$ (Bonferroni adjusted 0.05/12 coffee-CpGs)

Supplementary Table 10. Association of coffee-associated CpGs with smoking

CpG	β	P-value	β (smoking)	P-value (smoking)	PMID of papers
cg09935388	-0.0012	2.32E-08	-0.018	6.7E-11	PMID: 27651444
cg14476101*	0.0011	4.71E-08			
cg11550064	0.0007	2.11E-08	0.006	1.7E-09	PMID: 27651444
cg06690548*	0.0008	0.000000201			
cg21161138	-0.0011	6.66E-12	-0.008	1.6E-14	PMID: 27651444
cg23916896	-0.0013	4.76E-08	-0.017	1.9E-12	PMID: 27651444
cg25648203	-0.001	7.31E-14	-0.004	6.90E-08	PMID: 27651444
cg06126421	-0.0011	0.000000045	-0.036	3E-18	PMID: 27651444
cg15928106*	0.0015	1.59E-08			
cg20228731*	0.0015	3.87E-08			
cg05575921	-0.0016	2.17E-15	-0.04	8.2E-27	PMID: 27651444
cg03636183	-0.0014	1.15E-12	-0.026	8.9E-25	PMID: 27651444

* information on smoking has not been publicily reported

P-value < 0.0041 (Bonferroni adjusted 0.05/12 coffee-CpGs)

Supplementary Table 11. Association of coffee-CpGs with tea consumption

CpG	β	P-value
cg09935388	5.00E-04	0.1
cg14476101	4.00E-04	0.1
cg11550064	0	1
cg06690548	-2.00E-04	0.4
cg21161138	5.00E-04	0.01
cg23916896	4.00E-04	0.2
cg25648203	4.00E-04	0.03
cg06126421	4.00E-04	0.2
cg15928106	-5.00E-04	0.2
cg20228731	-6.00E-04	0.1
cg05575921	2.00E-04	0.4
cg03636183	5.00E-04	0.03

P-value < 0.0041 (Bonferroni adjusted 0.05/12 coffee-CpGs)
n= 15,789

Supplementary Table 12. IPA Pathway for genes annotated to coffee-associated CpGs

Top 3 canonical pathways		P-value
1	Serine Biosynthesis	1.36E-03
2	Superpathways of Serin and Glycine Biosynth I	1.90E-03
3	Xenobiotic Metabolism Signaling	2.71E-03
Top 3 dieases and disorders		P-value
1	Inflamatory Response	4.48E0-2, 4.42E-05
2	Organismal injury	4.41E0-2, 4.42E-05
3	Cancer	4.06E0-2, 2.72E-05
Top Tox Function		P-value
1	Increased levels of ALT	7.58E0-3

P-value treshold (P<0.05)

Supplementary Table 13. IPA Pathway for genes annotated to tea-suggestive associated CpGs

Top 3 canonical pathways		P-value
1	White adipose tissue browning	3.20E-05
2	nNOS signalling in skeletal muscle cells	3.60E-03
3	Maturity onset diabetes of young (MODY)	5.20E-03
Top 3 diseases and disorders		P-value
1	Developmental disorder	2.99E0-3, 3.98E-05
2	Organismal injury and abnormalities	4.87E0-2, 3.98E-05
3	Cardiovascular disease	3.36E0-2, 8.80E-05
Top Tox Function		P-value
1	Cardiac dilation	3.34E0-3, 8.80E-05

P-value treshold (P<0.05)

Supplementary Table 14. Correlation between coffee and tea-associated CpGs with SNPs in near-by (cis- meQTLs) or distant genes (TransmeQTLs), and with expression levels of nearby genes (cis-eQTMs)

CpG	Gene	CHR	CpG Chr. position	cis-meQTLs			cis-eQTMs			trans-meQTLs			
				SNP	SNP position	P-value	probe	probe position	P-value	SNP	CHR	SNP Chr. Position	P-value
Coffee consumption													
cg05575921	*EXOC3*	5	373402	rs6555226	389589	1.10E-16	ENSG00000180104	443273	1.19E-06				
				rs13152890	489598	2.42E-11							
				rs76312731	401734	5.60E-06							
cg25648203	*EXOC3*	5	395396	rs62331561	349256	2.20E-29	ENSG00000180104	443273	2.11E-13				
				rs2466287	420726	2.50E-20							
				rs73734213	303778	4.85E-10							
				rs7714003	540629	6.96E-05							
				rs11746373	491826	8.81E-05							
cg03636183	*F2RL3*	19	17000561	rs1054533	17004049	2.03E-45	ENSG00000127533	16999671	8.86E-06				
				rs2287794	16977060	1.16E-12							
cg21161138	*EXOC3*	5	399336	rs11746538	427466	1.85E-07	ENSG00000180104	443273	1.33E-04				
cg15928106		7	130646102	rs1059698	130629094	3.90E-17							
				rs969827	130640611	3.31E-09							
				rs10256380	130693931	8.20E-06							
cg11550064		2	240148167	rs291336	240148039	8.06E-32							
cg09935388		1	92947612	rs2046616	92831569	1.84E-18							
cg20228731		7	130646075	rs1059698	130629094	1.73E-16							
				rs969827	130640611	8.54E-10							
				rs10265812	130690824	1.11E-06							
cg06126421	*FLOT1*	6	30720056				ENSG00000137312	30710510	5.58E-07	rs11190133	10	101278725	1.75E-07
	LINC00243						ENSG00000214894	30798436	2.98E-05				
	TUBB						ENSG00000196230	30687978	6.97E-05				
cg14476101	*PHGDH*	1	120255968	rs11583993	120255370	3.85E-228	ENSG00000092621	120202421	2.05E-55				
				rs41276626	120262112	3.14E-14							
				rs34291690	120099137	9.22E-08							
cg23916896	NA												
cg06690548	*SLC7A11*	4											
cg01940273		2	233284910	rs2853384	233286652	2.69E-37							
		2	233284910	rs5013535	233234681	1.22E-05							
Tea consumption													
cg20099906		19	13344796	rs62109930	13275412	1.20E-04							

Abbreviations: CHR= chromosome; SE= standard error; NA= not available. P-values provided have been adjuted for multiple testing. More information can be found at https://www.genenetwork.nl/biosqtlbrowser/

Supplementary Table 15. Previously reported associations of coffee-associated CpGs with other phenotypes

CpG	CHR	Position	GeneID	Phenotype (PMID)
cg05575921	5	373378	*AHRR*	smoking (27651444), EA (29086770), BMI(25935004), CRP(27955697), WC (25935004), sex (26500701), alcohol consumption (27843151), HIV infection (27105112), gestational age (27717397), mortality (29419728), CVD (29326313), cognitive abilities (29311653), victimization stress (29325449), blood serum metabolic traits (24014485), atopy and atopic asthma (30584054), polychlorinated biphenyls and polychlorinated dibenzofurans (30640082), race-specific with metabolic syndrome (31739726), blood plasma protein traits (31900413), high blood pressure (31999706)
cg25648203	5	395444	*AHRR*	smoking (27651444), EA (29086770), alcohol consumption (27843151), mortality (29419728), cognitive abilities (29311653), victimization stress (29325449),race-specific with metabolic syndrome (31739726)
cg03636183	19	17000585	*F2RL3*	smoking (27651444), EA (29086770), alcohol consumption (27843151), mortality (29419728), cognitive abilities (29311653), victimization stress (29325449), blood serum metabolic traits (24014485), protein biomarkers (28915241), bisphenol A (31451752), race-specific with metabolic syndrome (31739726)
cg21161138	5	399360	*AHRR*	smoking (27651444), EA (29086770), CRP (27824951), HIV infection (27105112), alcohol consumption (27843151), victimization stress (29325449), polychlorinated biphenyls and polychlorinated dibenzofurans (30640082), race-specific with metabolic syndrome (31739726)
cg15928106	7	130646078	*FLJ43663*	sex (26500701), gestational age (27717397), clear cell renal carcinoma (23526956), pancreatic ductal adenocarcinoma (24500968), SETD1B- related syndrome (31685013), Chron's disease (30779925)
cg11550064	2	240148191	*HDAC4*	smoking (27651444), schizophrenia (27572077), Myasthenia Gravis (28549776), atopy and atopic asthma (30584054), multiple sclerosis (30479356)
cg09935388	1	92947588	*GFI1*	smoking (27651444), fetal vs adult liver (25282492), alcohol consumption (27843151), BMI(25935004), EA (29086770), WC (25935004), cognitive abilities (29311653),blood serum metabolic traits (24014485), victimization stress (29325449), oral cancer (28890207), psoriasis (30092825)

cg20228731	7	130646051	*FLJ43663*	Behçet's disease (30863869), clear cell renal carcinoma (23526956), sex (26500701), pancreatic ductal adenocarcinoma (24500968), gestational age (27717397), oral cancer (28890207), Chron's disease (30779925), SETD1B- related syndrome (31685013), severe sepsis (31833902)
cg06126421	6	30720080	NA	smoking (27651444), pancreatic ductal adenocarcinoma (24500968), CRP(27955697), EA (29086770), age (28811542), WC (25935004), alcohol consumption (27843151), sex (26500701), HIV infection (27105112), systemic lupus (29437559), mortality (29419728), cognitive abilities (29311653), victimization stress (29325449), blood serum metabolic traits (24014485), protein biomarkers (28915241), psoriasis (30092825)
cg14476101	1	120255992	*PHGDH*	liver enzymes (28624579), BMI (28002404), serum triglycerides (28213390), alcohol consumption (27843151), CRP(27955697), WC (26110892), sex (26500701), gestational age (27717397), adiposity (29713043), mortality (29419728), blood serum metabolic traits (24014485), blood pressure (29198723), atopy and atopic asthma (30584054), insuline resistance (30792424), incident T2D (31506343), SETD1B- related syndrome (31685013), hepatic fat (30936141)
cg23916896	5	368804	*AHRR*	smoking (27651444), EA (29086770), victimization stress (29325449)

* in case multiple EWASs replicated same CpG for same trait or similar traits
(eg. smoking, pack-years, maternal smoking, serum cotininine, lung function)
or in different populations, we reported the largest EWAS on the trait to avoid redundancy
EA = educational attainment; BMI = body mass index; WC = waist circumference;
CVD= cardiovascual disease

Supplementary Table 16. Mendelian randomization results between coffee consumption and cg14476101

Method	β	P-value	Pvalue heterogeneity (IVW)
IVW	0.01	0.1	0.9

Two-sample Mendelian randomization analysis done using 50 SNPs as Instrumental variables evaluated in 357,454 coffee drinkers. P-value < 0.05

Supplementary Table 17. Associations of CpGs-mQTLs with cardiometabolic traits by three different MR method

Trait			Type 2 diabetes			BMI			WHR			HDL			LDL			Total cholesterol			Triglycerides			CHD		
CpG	MR Method	Nr. of SNPS	Causal effect (SD)	P-value	Pval het	Causal effect (SD)	P-value	Pval het	Causal effect (SD)	P-value	Pval het	Causal effect (SD)	P-value	Pval het	Causal effect (SD)	P-value	Pval het	Causal effect (SD)	P-value	Pval het	Causal effect (SD)	P-value	Pval het	Causal effect (SD)	P-value	Pval het
cg01940273$_{mqtls}$	IVW	11	-1.2 (0.3)	0.0001	0.9	-0.2 (0.5)	0.0001	0.9	-0.2 (0.05	0.0001	0.5	-0.4 (0.09)	0.0001	1e-4	-0.6 (0.2)	0.0001	0.9	-0.9 (0.2)	0.0001	0.9	-0.3 (0.2)	0.04	0.9	-0.2 (0.2)	0.6	0.9
cg01940273$_{mqtls}$	Egger estimate	11	-3.7 (1.9)	0.06		-0.2 (0.3)	0.6		-1.2 (0.3)	0.0001					-0.2 (1.1)	0.8		-1.1 (1.1)	0.3							
cg01940273$_{mqtls}$	Egger intercept	11	0.04	0.2		0	0.9		0	0.003					-0	0.7		0	0.9							
cg01940273$_{mqtls}$	WM	11	-1.3 (0.4)	0.0001		-0.2 (0.06)	0.002		-0.2 (0.07)	0.0001					-0.6 (0.2)	0.005		-0.1 (0.2)	0.0001							
cg05575921$_{mqtls}$	IVW	9	-0.2 (0.2)	0.2	0.9	-0.1 (0.3)	0.0001	0.3	-0.1 (0.04)	0.001	0.9	-0.4 (0.1)	0.002	0.9	0.2 (0.1)	0.2	0.4	0.2 (0.1)	0.04	0.3	0.2 (0.1)	0.05	0.9	-0.009 (0.1)	0.8	0.5
cg05575921$_{mqtls}$	Egger estimate	9				-0.2 (0.1)	0.09		-0.02 (0.1)	0.9		-0.1 (0.5)	0.8		0.9 (0.5)	0.03		0.2 (0.5)	0.6		0.5 (0.4)	0.3				
cg05575921$_{mqtls}$	Egger intercept	9				0	0.4		-0	0.4		-0	0.6		-0	0.08		0	0.9		-0	0.6				
cg05575921$_{mqtls}$	WM	9				-0.2 (0.05)	0.001		-0.08 (0.05)	0.1		-0.3 (0.1)	0.02		0.3 (0.2)	0.07		0.2 (0.2)	0.1		0.3 (0.1)	0.05				
cg09935388$_{mqtls}$	IVW	37	-0.5 (0.08)	0.0001	0.5	-0.03 (0.03)	0.3	1e-4	-0.1 (0.2)	0.0001	1e-4	-0.06 (0.09)	0.5	1e-4	0.2 (0.09)	0.03	1e-4	0.2 (0.1)	0.3	1e-4	0.1 (0.06)	0.03	0.9	-0.2 (0.05)	0.001	0.9
cg09935388$_{mqtls}$	Egger estimate	37	-0.5 (0.1)	0.0001																				-0.12 (0.07)	0.1	
cg09935388$_{mqtls}$	Egger intercept	37	0	0.9																				-0	0.2	
cg09935388$_{mqtls}$	WM	37	-0.4 (0.1)	0.003																				-0.09 (0.08)	0.3	
cg11550064$_{mqtls}$	IVW	85	0.9 (0.3)	0.0001	1e-4	0.2 (0.04)	0.0001	0.8	0.9 (0.04)	0.0001	0.9	1.2 (0.1)	0.0001	0.9	1.9 (0.1)	0.0001	0.9	2.8 (0.1)	0.0001	0.9	1.8 (0.2)	0.0001	0.9	-0.8 (0.1)	0.0001	0.9
cg11550064$_{mqtls}$	Egger estimate	85				0.8 (0.2)	0.0001		0.8 (0.2)	0.0001		1.5 (0.6)	0.01		0.7 (0.6)	0.2		2.3 (0.6)	0.0001		1.9 (0.6)	0.001		0.5 (0.6)	0.4	
cg11550064$_{mqtls}$	Egger intercept	85				-0	0.001		0	0.4		-0	0.7		0	0.05		0	0.4		-0	0.6		0	0.03	
cg11550064$_{mqtls}$	WM	85				0.4 (0.05)	0.0001		0.9 (0.05)	0.0001		1.2 (0.2)	0.0001		1.8 (0.2)	0.0001		2.7 (0.2)	0.0001		1.8 (0.2)	0.0001		-0.8 (0.2)	0.0001	
cg14476101$_{mqtls}$	IVW	7	0.04 (0.3)	0.9	0.9	0.1 (0.06)	0.04	0.9	0.07 (0.06)	0.2	0.9	-0.4 (0.2)	0.08	0.8	0.3 (0.2)	0.1	0.9	0.2 (0.2)	0.4	0.9	0.6 (0.2)	0.006	0.8	-0.008 (0.2)	0.9	0.9
cg14476101$_{mqtls}$	Egger estimate	7																			-0.5 (0.7)	0.5				
cg14476101$_{mqtls}$	Egger intercept	7																			0	0.12				
cg14476101$_{mqtls}$	WM	7																			0.7 (0.3)	0.01				
cg15928106$_{mqtls}$	IVW	3	0.9 (0.4)	0.02	0.8	0.06 (0.07)	0.4	0.01	-0.1 (0.07)	0.09	0.9	0.3 (0.2)	0.2	0.8	0.3 (0.2)	0.1	0.3	0.5 (0.2)	0.02	0.1	0.1 (0.2)	0.5	0.2	0.2 (0.3)	0.3	0.3
cg15928106$_{mqtls}$	Egger estimate	3																								
cg15928106$_{mqtls}$	Egger intercept	3																								
cg15928106$_{mqtls}$	WM	3																								
cg23916896$_{mqtls}$	IVW	66	-0.2 (0.1)	0.1	0.01	-0.05 (0.02)	0.03	0.01	-0.1 (0.02)	0.0001	1e-4	-0.08 (0.1)	0.4	1e-4	0.4 (0.06)	0.0001	0.7	0.5 (0.07)	0.0001	0.005	-0.3 (0.06)	0.0001	0.9	-0.3 (0.07)	0.0001	0.9
cg23916896$_{mqtls}$	Egger estimate	66													0.1 (0.1)	0.5		-0.2 (0.2)	0.2		-0.6 (0.1)	0.0001		-0.7 (0.2)	0.0001	
cg23916896$_{mqtls}$	Egger intercept	66													0	0.05		0	0.0001		0	0.03		0	0.01	
cg23916896$_{mqtls}$	WM	66													0.6 (0.09)	0.0001		0.8 (0.1)	0.0001		-0.1 (0.09)	0.3		-0.2 (0.1)	0.02	

Casual association estimates between coffee-related CpGs and cardiometabolic traits.

CpG= DNA methylation site; CHR = chromosome, Pval het= P-value heteregoneity

Supplementary Table 18. Association of coffee consumption with serum levels of liver enzymes in the RS

	GGT		ALT		AST	
n= 4,756	β	P-value	β	P-value	β	P-value
coffee consumption	-0.011	0.0048	-0.005	0.051	-0.005	0.0083

Model adjusted for age, sex, BMI, smoking and excessive alcohol consumption.

Excessive alcohol consumption was defined as >14 units/week for women and >21 units/week for men, where the unit would correspond to 10 grams.

P value treshold = 0.016, adjusted for multiple testing of coffee with 3 liver enzymes (Bonferroni adjusted 0.05/3)
