# Supplementary material for: Epigenome-wide association meta-analysis of DNA methylation with coffee and tea consumption
Source: Nat Commun. 2021 May 14;12:2830. doi: 10.1038/s41467-021-22752-6 (PMC8121846; doi:10.1038/s41467-021-22752-6)
Supplement: Supplementary file 3 — Reporting Summary [file 41467_2021_22752_MOESM3_ESM.pdf]

nature research

Corresponding author(s): Mohsen Ghanbari

Last updated by author(s): Dec 30, 2020

Reporting Summary

Nature Research wishes to improve the reproducibility of the work that we publish. This form provides structure for consistency and transparency in reporting. For further information on Nature Research policies, see Authors & Referees and the Editorial Policy Checklist .

Statistics

For all statistical analyses, confirm that the following items are present in the figure legend, table legend, main text, or Methods section.

n/a	Confirmed	
☐	☒	The exact sample size (*n*) for each experimental group/condition, given as a discrete number and unit of measurement
☐	☒	A statement on whether measurements were taken from distinct samples or whether the same sample was measured repeatedly
☐	☒	The statistical test(s) used AND whether they are one- or two-sided<br>*Only common tests should be described solely by name; describe more complex techniques in the Methods section.*
☐	☒	A description of all covariates tested
☐	☒	A description of any assumptions or corrections, such as tests of normality and adjustment for multiple comparisons
☐	☒	A full description of the statistical parameters including central tendency (e.g. means) or other basic estimates (e.g. regression coefficient) AND variation (e.g. standard deviation) or associated estimates of uncertainty (e.g. confidence intervals)
☐	☒	For null hypothesis testing, the test statistic (e.g. *F*, *t*, *r*) with confidence intervals, effect sizes, degrees of freedom and *P* value noted<br>*Give P values as exact values whenever suitable.*
☒	☐	For Bayesian analysis, information on the choice of priors and Markov chain Monte Carlo settings
☒	☐	For hierarchical and complex designs, identification of the appropriate level for tests and full reporting of outcomes
☐	☒	Estimates of effect sizes (e.g. Cohen's *d*, Pearson's *r*), indicating how they were calculated

Our web collection on statistics for biologists contains articles on many of the points above.

Software and code

Policy information about availability of computer code

Data collection	The Illumina® Infinium HumanMethylation450 BeadChip and the Illumina's MethylationEPIC '850K' BeadChipthe were used for DNA methylation data.
Data analysis	R (V.1.2-2 and later) including QCEWAS, metaviz (V.0.3.1, https://CRAN.R-project.org/package=metaviz), and qqman packages (V.0.1.2, https://cran.r-project.org/web/packages/QCEWAS/index.html); METAL (V.2011-03-25, https://genome.sph.umich.edu/wiki/METAL_Documentation); GWAMA (V.2.2.2, https://genomics.ut.ee/en/tools/gwama); GenomeStudio (V.2011.1, https://emea.support.illumina.com/array/array_software/genomestudio/downloads.html); IPA software (V.01-12, https://www.qiagenbioinformatics.com/products/ingenuity-pathway-analysis/); MR-PRESSO (V.3.3.1, Mendelian Randomization pleiotropy residual sum and outlier (https://github.com/rondolab/MR-PRESSO); MendelianRandomization R-package (V.0.3.0, https://cran.rproject.org/web/packages/MendelianRandomization/index.html); Houseman algorithm (V2.0 , RefFreeEWAS, https://cran.rproject.org/web/packages/RefFreeEWAS/index.html). All the softwares are freely available through the links.

For manuscripts utilizing custom algorithms or software that are central to the research but not yet described in published literature, software must be made available to editors/reviewers. We strongly encourage code deposition in a community repository (e.g. GitHub). See the Nature Research guidelines for submitting code & software for further information.

Data

Policy information about availability of data

All manuscripts must include a data availability statement. This statement should provide the following information, where applicable:

- Accession codes, unique identifiers, or web links for publicly available datasets
- A list of figures that have associated raw data
- A description of any restrictions on data availability

The datasets generated during this study are available from the corresponding author upon reasonable request. The dataset used to extract genetic variants and gene expression data is based on the BIOS-BBMRI database (freely available at: http://www.genenetwork.nl/biosqtlbrowser/). Functional mapping and annotation was performed using FUMA GWAS (freely available at https://fuma.ctglab.nl/), which also includes GTEx data. All the software and programmes used to conduct these analyses are freely available through the links mentioned in the manuscript.

Field-specific reporting

Please select the one below that is the best fit for your research. If you are not sure, read the appropriate sections before making your selection.

☒ Life sciences ☐ Behavioural & social sciences ☐ Ecological, evolutionary & environmental sciences

For a reference copy of the document with all sections, see nature.com/documents/nr-reporting-summary-flat.pdf

Life sciences study design

All studies must disclose on these points even when the disclosure is negative.

Sample size	A total of 15 cohorts (N=15,789) mainly from the Epigenetics working group in the Cohorts for Heart and Aging Research in Genomics Epidemiology (CHARGE) consortium participated in this study. There was no a priori selection criteria for this study apart that collaborative cohorts had data available required for these analysis (dietary data, DNA methylation and confounders used in the main EWAS analysis). This resulted in 12,868 participants with European ancestry and 2,921 participants with African-American ancestry. We believe our study provides the largest epigenome-wide association studies (EWAS) of coffee and tea consumption.
Data exclusions	We excluded herbal tea and others, as green and black tea are derived from the different processing and harvesting of leaves from the same plant -Camellia sinensis (where applicable).
Replication	EWAS meta-analysis of 9,612 participants in the discovery phase identified 11 CpGs associated with coffee consumption. Seven of these CpGs were successfully replicated in independent cohorts from both ancestries (EA and AA) comprising 6,177 participants. This could be due to lower statistical power from smaller sample sizes and ethnic specific differences. The combined meta-analysis from the whole samples resulted in 11 CpGs significantly associated with coffee consumption at the epigenome-wide significance threshold (P < 1.1×10-7).
Randomization	This study is an observational study investigating the association between DNA methylation levels and coffee/tea consumption. So, we did not apply any randomization.
Blinding	As this is an observational study, there was no blinding. In this study, the phenotype of interest (coffee and tea consumption) need to be known in advance in order to conduct the EWAS analysis in each contributing cohort.

Reporting for specific materials, systems and methods

We require information from authors about some types of materials, experimental systems and methods used in many studies. Here, indicate whether each material, system or method listed is relevant to your study. If you are not sure if a list item applies to your research, read the appropriate section before selecting a response.

Materials & experimental systems

n/a	Involved in the study	
☒	☐	Antibodies
☐	☒	Eukaryotic cell lines
☒	☐	Palaeontology
☒	☐	Animals and other organisms
☐	☒	Human research participants
☒	☐	Clinical data

Methods

n/a	Involved in the study	
☒	☐	ChIP-seq
☒	☐	Flow cytometry
☒	☐	MRI-based neuroimaging

Eukaryotic cell lines

Policy information about cell lines

Cell line source(s)	SNU398, SNU449 and PLC/PRF/5 were originally provided by François Helle (Centre Universitaire de Recherche en Santé, Amiens, France). Huh6 was provided by Dr. Volker Lohmann from University of Heidelberg, Heidelberg, Germany. HepRG was ordered from Lonza. Hep3B and HEK293T were from ATCC.
Authentication	Identity of all cell lines was confirmed by Short Tandem Repeats (STRs) genotyping, performed at department of Pathology, Erasmus Medical Center Rotterdam.
Mycoplasma contamination	The cell lines were regularly tested for mycoplasma contamination, and were all confirmed as negative.
Commonly misidentified lines (See ICLAC register)	No commonly misidentified cell lines were used in this study.

Human research participants

Policy information about studies involving human research participants

Population characteristics	This study included a total of 15,789 participants in two phases. The discovery phase included 9,612 participants of European ancestry from Airwave, Avon Longitudinal Study of Parents and Children (ALSPAC), two independent datasets from the ESTHER Study (ESTHER_a and ESTHER_b), Framingham Heart Study (FHS), Cooperative Health Research in the Augsburg Region Study (KORA), two cohorts of the Rotterdam Study (RS-II and RS-III) and TwinsUK. The replication phase consisted of 6,177 participants of European (EA) and African American (AA) ancestries from two ethnically different sub cohorts of Atherosclerosis Risk in Communities Study (ARIC_EA and ARIC_AA), two ethnically different sub cohorts from the Cardiovascular Health Study (CHS_EA and CHS_AA), and two independent studies from the European Prospective Investigation into Cancer and Nutrition (EPIC_Italy and EPIC_IARC). The mean age across all participating cohorts ranged from 41.06 years in Airwave cohort to 78.6 years in CHS_EA cohort. Majority of the study participants were females 61.44%. Mean coffee intake ranged from 0.6 cups/day in CHS_AA cohort to 3.52 cups/day in RS-III-2 cohort, while tea intake ranged from 0.29 cup/day in EPIC_Italy cohort to 3.35 cups/day in Twins UK
Recruitment	This study was conducted within the framework of the Cohort for Heart and Aging Research in Genomic Epidemiology (CHARGE) consortium and additional participating cohorts, resulting in a total sample of 15,789 participants. Participating cohorts are Airwave, Avon Longitudinal Study of Parents and Children (ALSPAC), two independent datasets from the ESTHER Study (ESTHER_a and ESTHER_b), Framingham Heart Study (FHS), Cooperative Health Research in the Augsburg Region Study (KORA), two cohorts of the Rotterdam Study (RS-II and RS-III) and TwinsUK (in the discovery phase with European ancestry). Moreover, two ethnically different sub cohorts of Atherosclerosis Risk in Communities Study (ARIC_EA and ARIC_AA), two ethnically different sub cohorts from the Cardiovascular Health Study (CHS_EA and CHS_AA), and two independent studies from the European Prospective Investigation into Cancer and Nutrition (EPIC_Italy and EPIC_IARC) (in the replication phase with European and African American ancestries). More information about the participating cohorts are provided in the Supplementary material.
Ethics oversight	All 15 contributing cohorts confirmed compliance with their local research ethics committees or Institutional Review Boards for this study. Statement is included in the main text and also in the supplementary methods.

Note that full information on the approval of the study protocol must also be provided in the manuscript.
